# Nanomechanics of graphene

**DOI:** 10.1093/nsr/nwy067

**Published:** 2018-06-19

**Authors:** Yujie Wei, Ronggui Yang

**Affiliations:** 1 The State Key Laboratory of Nonlinear Mechanics (LNM), Institute of Mechanics, Chinese Academy of Sciences, Beijing 100190, China; 2 School of Engineering Sciences, University of Chinese Academy of Sciences, Beijing 100049, China; 3 Department of Mechanical Engineering, University of Colorado, Boulder, CO 80309, USA

**Keywords:** graphene, strength, wrinkling, pentagon–heptagon rings, carbon honeycomb

## Abstract

The super-high strength of single-layer graphene has attracted great interest. In practice, defects resulting from thermodynamics or introduced by fabrication, naturally or artificially, play a pivotal role in the mechanical behaviors of graphene. More importantly, high strength is just one aspect of the magnificent mechanical properties of graphene: its atomic-thin geometry not only leads to ultra-low bending rigidity, but also brings in many other unique properties of graphene in terms of mechanics in contrast to other carbon allotropes, including fullerenes and carbon nanotubes. The out-of-plane deformation is of a ‘soft’ nature, which gives rise to rich morphology and is crucial for morphology control. In this review article, we aim to summarize current theoretical advances in describing the mechanics of defects in graphene and the theory to capture the out-of-plane deformation. The structure–mechanical property relationship in graphene, in terms of its elasticity, strength, bending and wrinkling, with or without the influence of imperfections, is presented.

## INTRODUCTION

There exist many reviews on the synthesis, properties and applications of graphene, and it has not been our purpose merely to add one to the many. Our aim is to demonstrate the mechanics of graphene as an integral part of materials and structures other than the isolated domain of remarkable individuals. This purpose demands more account of general mechanical analysis. We have found that this is particularly necessary as a general reader cannot be assumed to be comfortable with the applicability of classical mechanics theories to the mechanics of graphene.

Until the success of Geim and co-workers in producing monolayer graphene by the mechanical exfoliation of graphite [1,2], atomically thin materials were believed to be thermodynamically unstable under ambient conditions [3,4]. The emergence of graphene leads to great attention being paid to examining its specialty in contrast to other carbon allotropes including fullerene, carbon nanotube (CNT) and graphite [5]. Among nearly 356 carbon allotropes from 500 scientific reports [6], the aforementioned carbon structures including fullerene, carbon nanotube and graphene are composed of a single layer of carbon atoms. As both carbon nanotubes and graphene are composed of sp^2^-bonded carbon atoms packed in a honeycomb crystal lattice, it is no surprise that graphene also has as extremely high modulus and high strength as the well-studied carbon nanotubes [7–9]. What makes graphene distinct from carbon nanotubes is the 2D structure and it can be synthesized as large-area flat sheets, which opens up many applications for which carbon nanotubes cannot be conveniently utilized.

The atomic-thin nature of graphene leads to its ultra-low bending rigidity, making it ‘soft’ for out-of-plane deformation. Graphene can be easily bent to form rich 3D morphology [10–12] under either mechanical or thermal undulation. The bending properties accounting for the rippling of graphene have been investigated. Also, due to the increased surface area, the interaction between graphene and other materials has been studied. Typical mechanical behaviors involving surface interaction including adhesion [13,14], peeling during transfer [15–17], as well as frictional behavior [18–21] have drawn the attention of broad engineering communities. Small 2D structures have edges, and those edges introduce mechanical complications and excitements [22]. Large-area graphene usually consists of patches of small-area graphene with different size and orientation, coupling with grain boundaries (GBs) and other defects [23–27]. The mechanics of those typical defects in graphene and their influence on the strength are also covered in this review. In short, the aim of this review article is to provide a comprehensive summary of the up-to-date progress on the mechanics of graphene and, more importantly, how to understand the relationship between structures and mechanical properties, with and without defects.

There are already several review papers in the literature covering different aspects of the mechanics of 2D materials. For example, the fracture of graphene has been reviewed by Teng *et al.* [28] where the authors summarized the recent progress in experimental and theoretical studies on the fracture behaviors of graphene, and also presented significant yet unresolved issues relating to the fracture of graphene. In the review by Cao *et al.* [29], the authors showed recent advances in the characterization of the mechanical behavior of atomically thin films. The review by Ivanovskii [30] generalized the information on the modification of graphene-based carbon materials through the creation of structural defects, introduction of substitutive impurities, adsorption of impurity atoms and mechanical deformations. Several mechanical properties including elastic constants, strength, friction and fracture of graphene have been included. A particular review on the tribology of graphene was given by Penkov *et al.* [31]. Recently, Akinwande *et al.* [32] reviewed the mechanical properties of graphene and 2D materials. Penev *et al.* [33] reviewed the development of theoretical and computational models on the role of certain transcending concepts such as elastic instabilities, dislocations and edges. CastroNeto *et al.* published a review paper [34] on the unusual 2D Dirac-like electronic behavior of graphene, where the effects of electron–electron and electron–phonon interactions in single-layer and multi-layer graphene are presented. Ando [35] gave a review on the characteristic features of electronic states and electrical transport in both graphene and carbon nanotubes. A series of review papers on the chemical properties of typical 2D materials can be found in [36–39] and references therein. Wu *et al.* [40] reviewed up-to-date advances in graphene-based micro-electrochemical energy-storage devices that utilized the unique features of graphene and foresaw the future development of graphene-based micro-supercapacitors. The thermal conductivity of 2D materials was reviewed by Gu *et al.* [41], where the effects of different physical factors, such as sample size, strain and defects, on thermal transport in 2D materials are summarized.

## ELASTICITY OF PRISTINE GRAPHENE

Because of the rich bonding types, different carbon allotropes show a large variety of elastic properties. Even being the same type of honeycomb lattice of carbon atoms, graphene and carbon nanotubes can exhibit quite different mechanical behaviors, as they are categorized as typical examples of 2D and 1D materials, respectively. In particular, the difference in the dimensionality of those allotropes gives rise to different degrees of freedom in response to external stimuli. For instance, while the in-plane elastic properties of pristine graphene could be largely inferred from that of the well-studied CNTs, the out-of-plane deformation mode in graphene requires a different theoretical tool. Here we introduce the available theoretical approaches to analyse the elastic response in graphene.

### 2D long-range crystalline order

For a long time, 2D crystals have been conceived of as structurally unstable because of long wavelength fluctuations according to the Mermin–Wagner theorem [42]. 2D crystals are extremely flexible and are prone to structural instability, which gives rise to microscopic corrugations of a graphene sheet [3,4]. Nearly one century ago, Peierls [43] and Landau [44] concluded that there can be no 1D or 2D long-range crystalline order. The argument given by Peierls is qualitative based on the harmonic interaction of atoms in the 1D chain. Landau's conclusion on the non-existence of a 2D long-range crystalline order is based on his general theory of second-order phase transition [44], which is known to be misleading near the critical point. Alder and Wainright investigated a 2D system consisting of 870 particles placed in a periodically rectangular array. Their computer experiments, however, have indicated a transition to a 2D crystalline ordered state [45]. The theoretical analysis by Mermin a few years later [3] excluded a long-range crystalline order in two dimensions for power-law potentials of the Lennard-Jones type, but it is inconclusive for other types of potentials. The conclusion was made by deriving the Fourier component of the density for each vector of the reciprocal lattice. Considering a 2D crystalline flake with the Bravais lattice ascribed by ${\boldsymbol{\vec{a}}}$ and ${\boldsymbol{\vec{b}}}$ where the flake sides are defined by ${N_1}{\boldsymbol{\vec{a}}}$, ${N_2}{\boldsymbol{\vec{b}}}$, an arbitrary lattice point is defined by ${\boldsymbol{\vec{r}}} = m{\boldsymbol{\vec{a}}} + n{\boldsymbol{\vec{b}}}$, $m = 0,1, \ldots , {N_1}\ {\rm and}\ n = 0, 1,\ldots , {N_2} $. The total number of atoms in the flake is then $N = c{N_1}{N_2}$, where $c$ is the number of atoms in the unit cell defined by ${\boldsymbol{\vec{a}}}$ and ${\boldsymbol{\vec{b}}}$. The real space density distribution is simply given by

(1) \begin{equation*} \hat{\rho }\left( {{\boldsymbol{\vec{r}}}} \right) = \sum\nolimits_{i = 1}^N \delta \left({{\boldsymbol{\vec{r}}} - {{{\boldsymbol{\vec{r}}}}_i}}\right). \end{equation*}

In the reciprocal space, the *k*-th component of the Fourier's transform of the real space is obtained as

(2) \begin{equation*} \hat{\rho }\ \big( {\boldsymbol{\vec{k}}} \big) = \smallint \hat{\rho }\left( {{\boldsymbol{\vec{r}}}} \right){{\rm{e}}^{ - {\rm{i}}{\boldsymbol{\vec{k}}} \cdot {\boldsymbol{\vec{r}}}}}d{\boldsymbol{\vec{r}}} = \sum \nolimits_{i = 1}^N {{\rm{e}}^{ - {\rm{i}}{\boldsymbol{\vec{k}}} \cdot {{{\boldsymbol{\vec{r}}}}_i}}}. \end{equation*}

The integration is over all the atoms in the flake. By defining the *k*-th Fourier's component of the density

(3) \begin{equation*} {\rho _{{\boldsymbol{\vec{k}}}}} = \frac{1}{N} \big\langle \hat{\rho }\big( {\boldsymbol{\vec{k}}} \big) \big\rangle, \end{equation*}

with $\langle \cdots\rangle$ an averaging of density in the reciprocal space

(4) \begin{eqnarray*} \big\langle \hat{\rho } \big( {\boldsymbol{\vec{k}}} \big)\big\rangle \! =\!\! \smallint\! \frac{{\hat{\rho }\big( {{\boldsymbol{\vec{k}}}} \big){{\rm{e}}^{ - {\rm{U}} \left( {{{{\boldsymbol{\vec{r}}}}_1},{{{\boldsymbol{\vec{r}}}}_2}, \ldots ,{{{\boldsymbol{\vec{r}}}}_N}} \right)/kT}}d{{{\boldsymbol{\vec{r}}}}_1} \ldots d{{{\boldsymbol{\vec{r}}}}_N}}}{{\smallint {{\rm{e}}^{ - {\rm{U}}\left( {{{{\boldsymbol{\vec{r}}}}_1},{{{\boldsymbol{\vec{r}}}}_2}, \ldots ,{{{\boldsymbol{\vec{r}}}}_N}} \right)/kT}}d{{{\boldsymbol{\vec{r}}}}_1} \ldots d{{{\boldsymbol{\vec{r}}}}_N}}},\!\!\!\!\!\nonumber\\ \end{eqnarray*}

where $kT$ is the product of the Boltzmann constant and the absolute temperature with the unit Kelvin (K). Atoms in the flake interact through the pair potential ${\rm{\Phi }}( {{\boldsymbol{\vec{r}}}} )$, and we hence have the expression of internal energy ${\rm{U\ }}( {{{{\boldsymbol{\vec{r}}}}_1}, {{{\boldsymbol{\vec{r}}}}_2}, \ldots , {{{\boldsymbol{\vec{r}}}}_N}} ) = \frac{1}{2}\ \mathop \sum \nolimits_{i \ne j} {\rm{\Phi }}( {{{{\boldsymbol{\vec{r}}}}_i} - {{{\boldsymbol{\vec{r}}}}_j}} )$. For an entirely crystalline object, it stands to reason that there is an array of sharp peaks in the reciprocal space and ${\rho _{{\boldsymbol{\vec{k}}}}}$ will be non-zero. Mermin [3] adopted as a criterion for the existence of thermodynamically periodic arrangement of atoms in the flake the following: $\mathop {\lim }\nolimits_{{N_1}, {N_2} \to \infty } {\rho _{{\boldsymbol{\vec{k}}}}} \ne 0$ if ${\boldsymbol{\vec{k}}}$ is a reciprocal-lattice vector, and $\mathop {\lim }\nolimits_{{N_1}, {N_2} \to \infty } \ {\rho _{{\boldsymbol{\vec{k}}}}} = 0$ for other cases of ${\boldsymbol{\vec{k}}}$. He gave a proof that the first case cannot be satisfied in two dimensions for ${\rm{\Phi }}( {{\boldsymbol{\vec{r}}}} )$ of power-law potentials of the Lennard-Jones type, but it is inconclusive for other types of potentials [3]. In 2002, *ab-initio* calculations showed that a graphene sheet is thermodynamically unstable if its size is less than about 20 nm and it becomes the most stable fullerene only for molecules larger than 24,000 atoms [46].

The success of Geim and co-workers to produce monolayer graphene by the mechanical exfoliation of graphite [1,2] ended the theoretical controversy on thermodynamically stable 2D crystals under ambient conditions. Now many one-atom-thin and multi-atom-thin 2D materials were discovered with exploded literature studying their synthesis, physical properties and applications. While large-area pristine graphene may not be flat under thermal undulation [11], it can resist bending and remains 2D, in particular when the size of a graphene flake is small. It should be pointed out that sp^2^ bonding among carbon atoms in graphene certainly deviates from the power-law inter-atomic potential assumed in the theoretical analysis by Mermin [3]. In that sense, Mermin's analysis does not exclude the existence of stable 2D crystals with interactions significantly different from the Lennard-Jones type of potentials.

Very recently, Kumar and Parks [47] further analysed the lattice dynamic stability of graphene under straining and deduced a general continuum criterion for the onset of various kinds of lattice instabilities in graphene: an instability appears when the magnitude of the deviatoric strain $\gamma $ reaches a critical value ${\gamma _c}$, which depends on the mean normal strain and the directionality $\theta $ of the principal deviatoric stretch with respect to the reference lattice orientation. The criterion could be employed to distinguish fundamentally different mechanisms of lattice instabilities in graphene, such as structural versus material instabilities and long-wave (elastic) versus short-wave instabilities.

### In-plane elasticity

Given the atomic-thin nature, how 2D crystals resist deformation is of paramount interest. In this subsection, we first examine the in-plane elastic properties of single-layer graphene. As imagined, graphene is the mother element of some carbon allotropes including CNTs [48,49]. Naturally, the same lattice in graphene and CNT may be expected to have similar elasticity. The distinction originates from the structures. The elastic modulus of CNT is normally effective from the structure level: the stress is typically averaged over the circular cross-section of the tube. It is an effective modulus that may depend on the chirality of the CNT. For graphene, we consider the most general case regarding the mechanical response of graphene with the honeycomb lattice.

It is known that the change in the free energy $F$ in an anisotropic crystal under small deformation can be described by a quadratic function of the strain (in the Cartesian coordinate) [5]:

(5) \begin{equation*} F = \frac{1}{2}{c_{ijkl}}{\varepsilon _{ij}}{\varepsilon _{kl}}, \end{equation*}

where $i, j, k\ {\rm{and}}\ l$ are integers ranging from 1 to 3, and ${\varepsilon _{ij}}$ are strain components. The coefficient ${c_{ijkl}}$ is the rank-four elastic modulus tensor, including a maximum of 21 independent components for the triclinic system, but fewer in the crystals possessing symmetry. For hexagonal systems like graphene, their normal plane is the sixth-order axis: rotating through an angle of ${\rm{\pi }}/3$ about the plane normal results in the exact same lattice. The free energy is hence simplified as

(6) \begin{eqnarray*} F &=& \frac{1}{2}\ {c_{3333}}\varepsilon _{33}^2 + 2{c_{1212}}{\left( {{\varepsilon _{11}} + \,{\varepsilon _{22}}} \right)^2}\nonumber\\ && + \,{c_{1122}}\left[ {{{\left( {{\varepsilon _{11}} - {\varepsilon _{22}}} \right)}^2} + 4\varepsilon _{12}^2} \right] + 2{c_{1233}}{\varepsilon _{33}}\nonumber\\ && \times \,\left( {{\varepsilon _{11}} + {\varepsilon _{22}}} \right) + 4{c_{1223}}\left( {\varepsilon _{13}^2 + \varepsilon _{23}^2} \right). \end{eqnarray*}

From Equation (6), we can see that five independent elastic moduli are needed to describe the elastic deformation of a hexagonal crystal. When only the in-plane deformation is considered, namely ${\varepsilon _{i3}} = 0$ for $i = 1 \cdots 3$, the free energy is determined by the two elastic constants ${c_{1212}}$ and ${c_{1122}}$ in Equation (6). It hence suggests that the elastic behavior of 2D hexagonal crystals like graphene is isotropic in nature, consistently with other explorations [50].

**Figure 1.**
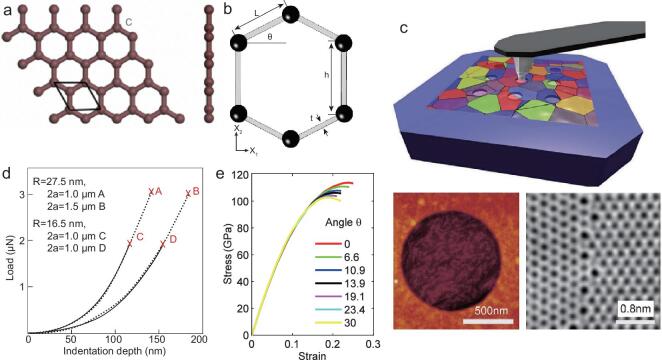
The stress–strain response of pristine 2D materials with the hexagonal lattice. (a) Graphene lattice. (b) Continuum approximation of a honeycomb structure. (c) Sketch of using nano-indentation to measure the strength of graphene GBs. The top shows the indenter on a polycrystalline graphene; bottom left shows the top view of a dent with graphene on the top and bottom right shows a graphene GB. Adapted from [9], with permission of Springer Nature. (d) Indenting load versus depth. The two sets of data demonstrated that the force-displacement response is independent of the tip radius of an indenter, and the breaking force marked at each point corresponds to different loading conditions. Adapted from [7], with permission of American Association for the Advancement of Science. (e) The stress–strain behavior of graphene loaded with different angle $\theta $ with respect to the zigzag edge. Adapted from [58], with permission of American Chemical Society Publications.

The isotropic elastic property of a hexagonal lattice can be alternatively understood at a macroscopic level if we take an analogy between the lattice and the honeycomb structure [51]. In the most general case, deformation in the ${X_1} - {X_2}$ plane in a honeycomb composed of unit cells shown in Fig. 1a and b is described by five moduli: two Young's moduli ${E_1}$ and ${E_2}$, the shear modulus of ${G_{12}}$ and two Poisson's ratios ${\nu _{12}}$ and ${\nu _{21}}$. But there are only four independent ones as the reciprocal relation requires ${E_1}\ {\nu _{21}} = {E_2}\ {\nu _{12}}$. The two moduli are thus given, respectively, as

(7a) \begin{equation*} \frac{{{E_1}}}{{{E_w}}} = {\left( {\frac{t}{L}} \right)^3}\ \frac{{\cos \theta }}{{\left( {h/L + \sin \theta } \right){{\sin }^2}\theta}} \end{equation*}

and

(7b) \begin{equation*} \frac{{{E_2}}}{{{E_w}}} = {\left( {\frac{t}{L}} \right)^3}\ \frac{{h/L + \sin \theta }}{{{{\cos }^3}\theta }}, \end{equation*}

where ${E_w}$ is the Young's modulus of the wall material. It is convenient to see that, for a hexagonal lattice, we have ${E_1} = {E_2}$. The Poisson's ratio, for loading along the ${X_1}$-direction, is given as

(7c) \begin{equation*} {\nu _{12}} = \frac{{{{\cos }^2}\theta }}{{\left( {h/L + \sin \theta } \right)\sin \theta }}, \end{equation*}

and that is

(7d) \begin{equation*} {\nu _{21}} = \frac{{\left( {h/L + \sin \theta } \right)\sin \theta }}{{{{\cos }^2}\theta }}\ \end{equation*}

when loading along the ${X_2}$-direction.

Again in a hexagonal lattice, we see $\nu = {\nu _{12}} = {\nu _{21}} = 1$ for $\theta \ = 30^\circ $ and $h/L = 1$. The shear modulus of the honeycomb is thus

(7e) \begin{equation*} \frac{{{G_{12}}}}{{{E_w}}} = {\left( {\frac{t}{L}} \right)^3}\ \frac{{h/L + \sin \theta }}{{{{\left( {h/L} \right)}^2}\left( {2h/L + 1} \right)\cos \theta }}.\end{equation*}

In the case of an ideal hexagon lattice, we also have the relation of ${G_{12}} = {\rm{E}}/2( {1 + {\rm{\nu }}} )$, which generally applies for isotropic solids. While there are apparent discrepancies between the hexagonal lattice like graphene and the hexagonal honeycomb as bending is considered to be the primary mechanism for elastic deformation for honeycombs, the comparison of the two systems can help us understand the isotropic nature of the in-plane mechanical properties in graphene under small deformation. When the deformation becomes large and the unit cell is severely distorted, changes in $\theta $ and $h/L$ would consequentially render the system to be anisotropic. This observation may explain the non-linear behavior of graphene during the elastic stage [52] and the slight anisotropic mechanical properties reported in the literature.

Aside from the topological difference, single-wall carbon nanotubes have the same lattice structure as that of graphene. Hence, the tensile behavior of graphene can essentially be deduced from those of carbon nanotubes, as long as the tube radius is not too small. For example, Van Lier *et al.* reported the Young's modulus and the Poisson ratio for a number of closed single-wall carbon nanotubes using the first all-electron *ab-initio* calculation [53]. The results agree well with those of graphene. Typical theoretical analysis predicts a Young's modulus of graphene to be higher than 1 TPa. The Poisson ratio in graphene is found to be small, close to 0.1 in a broad range of temperatures [54]. Liu *et al.* [52] calculated the phonon spectra of graphene as a function of the uniaxial tension by the density functional perturbation theory (DFPT) to assess the first occurrence of phonon instability on the strain path, which controls the strength of a defect-free crystal at 0 K. They obtained a Young's modulus *E* = 1050 GPa and Poisson's ratio *v* = 0.186 from small-strain calculations.

### In-plane nonlinearity

In the first effort to measure the break strength of graphene (Fig. 1c), Lee *et al.* [7] employed the indentation tests and found that the maximum stress attained in the film agreed well with an analytical solution [55] when a linear stress–strain curve is employed. To match the load-deflection curve as shown in Fig. 1d, however, the strains in the graphene near the indenter had to be set to values well over 0.2, which is far beyond the linear elastic regime. The stress–strain curves along different loading directions do show nonlinearity and large break strain (see Fig. 1e). Therefore, a non-linear elastic constitutive behavior [56] has to be adopted by counting the higher-order strain terms in the strain energy density formula [55]:

(8) \begin{equation*} F\ = \frac{1}{2}\ {c_{ijkl}}{\varepsilon _{ij}}{\varepsilon _{kl}} + \frac{1}{2}{D_{ijklmn}}{\varepsilon _{ij}}{\varepsilon _{kl}}{\varepsilon _{mn}}, \end{equation*}

where ${D_{ijklmn}}$ are the third-order elastic moduli. Casting this relationship in a uniaxial strain context, Lee *et al.* [7] wrote the stress, $\sigma $, and strain,$\ \varepsilon $, relationship as $\sigma = E\varepsilon + D{\varepsilon ^2}$. Such a relationship captures reasonably well the non-linear response of the indentation force versus depth curves from their experiments. The same technique was also adopted later for measurement of the strengths of the GBs in graphene [9] and graphene oxide [8]. Cadelano *et al.* [57] discussed the physical meaning of the effective non-linear elastic modulus. The authors developed the constitutive non-linear stress–strain relation for graphene and predicted its value to be in good agreement with available data in the literature. It is essential to use a physically sound constitutive model to capture the non-linear region in the stress–strain curves, although another issue—the anisotropic failure strength of graphene [58]—has been largely ignored in the existing analysis.

### Out-of-plane deformation

In contrast to its in-plane deformation, the out-of-plane deformation that is peculiar to the one-atom-thin graphene is of importance. Carbon atoms restricted to a finite-sized surface closure such as a sphere (fullerene) or a tube (CNT) can behave very differently from graphene flakes that are essentially flat. Free-standing graphene can easily be bent under thermal undulation [11]. Pivotal to this are the two mechanical parameters—bending rigidity and Gaussian bending stiffness—which govern the morphology of graphene under external stimuli. The structure and morphology manipulation, on the other hand, are broadly investigated for the potential applications of graphene in biological systems and stretchable electronics [59].

#### Bending stiffness

A reliable characterization of the bending rigidity and Gaussian bending stiffness of graphene is of significance for both the design and the manipulation of graphene morphology for engineering applications. It may be interesting to see first the prediction from the continuum mechanics, although there are always concerns about the applicability of the continuum theories for predicting the mechanical properties of graphene or carbon nanotubes (CNTs). Based on the Kirchhoff–Love theory, the bending stiffness of thin plates is determined as

(9) \begin{equation*} {B_M} = {\nu _{12}} = \frac{{E{H^3}}}{{12\left( {1 - {{\rm{\nu }}^2}} \right)}}, \end{equation*}

where $E$ is the Young's modulus, ${\rm{\nu }}$ is the Poisson's ratio and *H* is the thickness of the thin plate. For graphene, we have *E* = 1050 GPa and υ = 0.186 [60–62]. The thickness *H* is where most controversies originated from. If one extrapolates the graphene thickness by counting its stacking in the crystalline graphite, we have *H* = 0.34 nm. Substituting this number *H* = 0.34 nm into Equation (9) yields *B_M_* = 22.3 eV, which is significantly larger than the measured value [63]. The breakdown of the applicability of the continuum theory to predict the bending properties in graphene suggests that *H* may not be 0.34 nm for single-layer graphene while a smaller effective thickness might reconcile the difference for ${B_M}$ between the prediction by the continuum mechanics theory and the experimental value.

**Figure 2.**
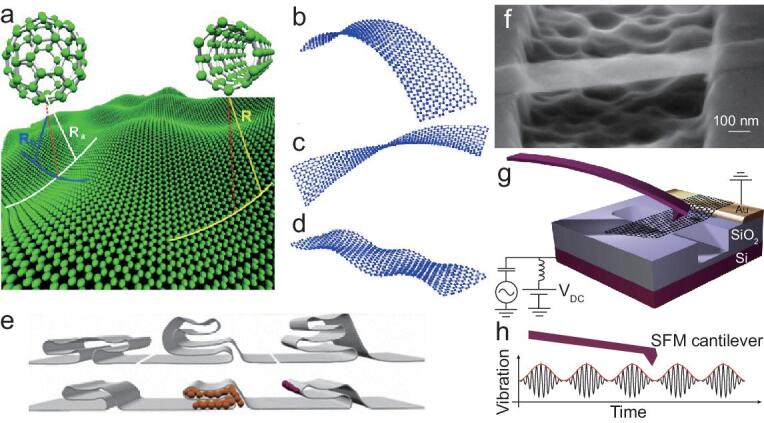
Governing parameters and modes of out-of-plane deformation of graphene. (a) The two governing material properties—bending rigidity and Gaussian bending stiffness—were deduced from single-wall carbon nanotubes and fullerenes of different sizes. Adapted from [59], with permission of American Chemical Society Publications. (b)–(d) Morphology of graphene nanoribbons resulted from edge stress-induced warping. Adapted from [91], with permission of American Physical Society. (e) Various complex folding structures. Adapted from [90], with permission of American Physical Society. (f)–(h) Mechanical vibrations in suspended graphene sheets. Adapted from [101], with permission of American Chemical Society Publications.

Instead of applying the continuum mechanics theory to calculate the bending stiffness of the low-dimensional carbon nanostructures, atomistic simulations have also been broadly used to predict the mechanical behaviors of small structures and systems. Wei *et al.* [59] applied the density functional theory (DFT) calculations to obtain the energies of 0D fullerenes and 1D single-wall carbon nanotubes (SWCNTs) of different radii and then derived both the bending rigidity and the Gaussian bending stiffness of free-standing single-layer graphene (Fig. 2a).

By combining the DFT calculations of energies of fullerenes and SWCNTs with the configurational energy of membranes determined by the Helfrich Hamiltonian [64,65], Wei *et al.* [59] designed a theoretical approach to accurately determine the bending rigidity and Gaussian bending stiffness of single-layer graphene. For a membrane with 3D topology, the configurational energy ${\psi _H}$ described by the Helfrich Hamiltonian [64,65] as

(10) \begin{eqnarray*} {\psi _H} = \mathop \smallint \nolimits_S\! \left[\! {\gamma + 2{B_M}{{\left( {{C_M} - {C_0}/2} \right)}^2} + {B_G}{C_G}}\! \right]ds ,\!\!\!\!\!\!\! \nonumber\\ \end{eqnarray*}

where $\gamma $ is the energy for unitary flat surface, ${B_M}$ is the flexural stiffness (bending rigidity), ${B_G}$ is the Gaussian bending stiffness, ${C_M} = ( {{k_1} + {k_2}} )/2$ is the mean curvature whereas ${k_1}$ and ${k_2}$ are the two principal curvatures of a 3D surface, ${C_G} = {k_1}\ {k_2}$ is the Gaussian curvature, and ${C_0}$ is the spontaneous curvature, which disappears for symmetrical membranes. The integral in Equation (10) extends over the whole surface.

Considering single-layer graphene as a membrane, the surface energy $\gamma $ remains constant during pure bending. By utilizing the fact that a SWCNT can be rolled up from a graphene [5], in graphene, the term associated with the Gaussian curvature disappears. By varying the radius of SWCNTs, one would then find that the energy per atom in SWCNT can be related to the energy per atom in graphene with the radii of SWCNTs by

(11) \begin{equation*}E_{atom}^{CNT} = {E_0} + {S_0}\frac{{{B_M}}}{{2{r^2}}}, \end{equation*}

where *r* is the radius of SWCNTs, $E_{atom}^{CNT}$is the energy per atom in a SWCNT, ${E_0}$ is the energy of an atom in a flat graphene and ${S_0} = 3\sqrt 3 {d^2}/4 = 2.63\,{\mathring{\rm A}^2}$ is the planar footprint (area) of a carbon atom in graphene with *d* being the C–C bond length. By calculating the energy per atom in SWCNTs with different radii, it is straightforward to derive the bending rigidity of graphene using Equation (11). To determine the Gaussian bending stiffness ${B_G}$, a series of spheroidal fullerenes with total atom numbers from 60 to 540 are considered, which have a corresponding radius variation from 1.86 to about 11 Å. Using Equation (10), the energy per atom in fullerenes can be given as

(12) \begin{equation*}E_{atom}^F = {E_0}\ + {S_0}\frac{{2{B_M} + {B_G}}}{{{r^2}}}.\end{equation*}

With ${B_M}$ known using Equation (11), ${B_G}$ in Equation (12) could then be determined. The bending rigidity and Gaussian bending stiffness of single-layer graphene are 1.44 (2.31 × 10^−19^ Nm) and −1.52 eV (2.43 × 10^−19^ Nm), respectively. The bending rigidity from this model is close to the measured result.

#### Correlation between the two stiffnesses

The relationship between the bending rigidity and the Gaussian bending stiffness in graphene is intriguing. The graphene sheet may be treated classically as an elastic plate and the solutions to the bending deformation of thin plates can be adopted. Assuming isotropic and homogeneous, and following Timoshenko and Woinowsky [66], the differential equation of the deflection $z$ of a graphene sheet is given as

(13) \begin{equation*} \frac{{{\partial ^4}z}}{{\partial {x^4}}} + 2\frac{{{\partial ^4}z}}{{\partial {x^2}\partial {y^2}}} + \ \frac{{{\partial ^4}z}}{{\partial {y^4}}} = \frac{q}{{{B_M}}}, \end{equation*}

where *q* is the distributed load normal to the surface. By neglecting the contribution of the shearing stress on the deflection of the graphene, its total energy is then given as

(14) \begin{eqnarray*} \!\!\!\!\!\!\psi &=& \frac{1}{2}\ {B_M}\smallint \left\{ {{\left( {\frac{{{\partial ^2}z}}{{\partial {x^2}}} + \frac{{{\partial ^2}z}}{{\partial {y^2}}}} \right)}^2} - 2\left( {1 - \nu } \right)\right.\nonumber\\ &&\times \left.\left[ {\frac{{{\partial ^2}z}}{{\partial {x^2}}}\frac{{{\partial ^2}z}}{{\partial {y^2}}} - {{\left( {\frac{{{\partial ^2}z}}{{\partial x\partial y}}} \right)}^2}} \right] \right\}dxdy,\quad \end{eqnarray*}

where the integration is extended over the entire surface of the graphene.

Recognizing that

(15a) \begin{equation*} \left( {{k_1} + {k_2}} \right) = - \left( {\frac{{{\partial ^2}z}}{{\partial {x^2}}} + \frac{{{\partial ^2}z}}{{\partial {y^2}}}} \right)\end{equation*}

and

(15b) \begin{equation*} \left( {{k_1}{k_2}} \right) = \frac{{{\partial ^2}z}}{{\partial {x^2}}}\ \frac{{{\partial ^2}z}}{{\partial {y^2}}} - {\left( {\frac{{{\partial ^2}z}}{{\partial x\partial y}}} \right)^2}, \end{equation*}

we may split the energy terms in Equation (14) into the bending and the twisting contributions, respectively. By substituting the two terms in Equation (15) into Equation (14) and then making a comparison with Equation (10), the following relationship between ${B_M}$ and ${B_G}$ for thin plates is obtained:

(16) \begin{equation*} \frac{{{B_G}}}{{{B_M}}} = \nu - 1.\end{equation*}

Recalling that the Poisson ratio in graphene is found to be small, approximate to 0.1 in a broad temperature range [54], we will have a $\frac{{{B_G}}}{{{B_M}}}$ ratio of about –0.9. If Equation (16) is applied to graphene, we find that the prediction roughly matches the results from DFT calculations [59].

In the classical theory, the value of an acceptable Poisson ratio should be positive. Yu and Ru [67] showed that, when the ratio of the Gaussian bending rigidity to the common flexural rigidity falls within the non-classical range, the actual mechanical behavior of such a membrane with two independent bending rigidities could be very sensitive to the exact values of the two independent bending rigidities and hence the Poisson ratio. Recently, Davini *et al.* [68] considered a discrete model of a graphene sheet with inter-atomic interactions governed by the harmonic approximation of the second-generation Brenner potential, which depends on bond lengths, bond angles and two types of dihedral angles. The authors proposed an analytical expression for the Gaussian stiffness, which turns out to be in good agreement with the DFT calculations. It was revealed recently [69] that the thermal fluctuations of elastic sheets can affect the effective bending stiffness at finite temperatures. Zhang *et al.* [70,71] demonstrated controllable out-of-plane wrinkles by utilizing the ultra-low bending stiffness of graphene and topological defects. Boddeti *et al.* [72] demonstrated that graphene blisters with switchable shapes could be realized by controlling pressure and adhesion, where both stiffnesses play a governing role for morphology stability.

### Edge effects

One of the most significant geometrical features of graphene is the unprecedented surface-area-to-volume ratio owing to the nature of one-atom thickness. The ultra-low bending resistance of such thin layers makes it difficult to control their morphology. Furthermore, the presence of edges in graphene nanostructures gives rise to rich morphology change due to the non-equilibrium edge atoms. With an aberration-corrected transmission electron microscope with both atomic-scale spatial resolution and 1-second temporal resolution, Girit *et al.* [73] observed the dynamics of carbon atoms at the edge of single-layer graphene. The detailed edge reconstruction and the stability of the ‘zigzag’ edge configuration were reported. The results of the *ab-initio* calculations of the effect of reconstruction and passivation of zigzag edges demonstrated that hydrogen-passivated ideal zigzag edges are more energetically favored than the pentagon–heptagon zigzag edges. However, the reconstructed edge is more stable in the absence of hydrogen [74]. At high temperature and low (quasi-static) mechanical loading rate [75], it is possible to obtain fully reconstructed zigzag edges through sequential reconstructions at the crack tip during the fracture of graphene.

The success in making graphene ribbons (GNRs) with nanoscale widths on the order of tens of nanometers [76–80] boosts the research in understanding the morphologies of those nanostructures [81], in addition to the theoretical predictions that the edge states in GNRs lead to the size effects on the electronic state of graphene for being metallic, insulating or semiconducting [82,83]. Point defect annealing and edge reconstruction during the edge-reconstruction process lead to ribbon morphology patterning and distinct physical properties resulting from the local edge structure [80]. When the electron and phonon mean free paths are comparable to or even greater than the ribbon widths, electron and phonon transport can be altered dramatically [84–86]. A number of studies reported ballistic, hydrodynamical and even rectified phonon transport in graphene nanoribbons after Hu *et al.* [87]. Ritter and Lyding [88] used tunneling spectroscopy to show that the electronic structure of GNRs with 2- to 20-nm width varies on the basis of graphene edge lattice symmetry. GNRs with a higher fraction of zigzag edges exhibit a smaller energy gap than a predominantly armchair-edge ribbon of a similar width. Zhang *et al.* [89] reviewed the chemical properties in graphene edges, especially the catalyst-passivated graphene edges and their role in graphene chemical vapor deposition (CVD) growth. Kim *et al.* [90] experimentally demonstrated that folded structures in graphene could be realized by introducing anisotropic surface curvature during graphene synthesis or material transfer processes, and this consequentially modifies the electronic band structure of graphene.

Distinct bonding characteristics of atoms at the free edges of GNR may introduce excessive edge energy and edge force that are also chiral-dependent [91–96]. Shenoy *et al.* [91] demonstrated that edge stresses could introduce intrinsic ripples in a free-standing graphene sheet even in the absence of any thermal effects. Compressive edge stresses along the zigzag and the armchair edges of the sheet can cause out-of-plane warping to attain several degenerate shape modes. Cranford and Buehler [97] developed a mesoscopic 2D model for a graphene sheet utilizing coarse-grain bead-spring elements with rotational-spring potentials. The authors used their mesoscopic model to study the structure and conformational behavior of twisted ultra-long multi-layer graphene ribbons with lengths of hundreds of nanometers. They even revealed a distinct transition from a twisted (saddle-like) configuration to a helical (coil-like) configuration as a function of the imposed rotation and number of graphene layers.

Deformation of GNRs is driven by the excess energy in the presence of edges and the subsequent edge stress. The mechanical deformation of a free-standing GNR could be modeled as a long membrane, with edges represented by elastic strings either in an elongated or compressed state and glued to the long edges of the membrane. Taking an analogue between the edge stress of a 2D membrane and surface stress of a 3D bulk solid, Shenoy *et al.* [91] wrote the energy per unit length of a GNR with width $W$ as

(17) \begin{equation*} {\cal F}\ \left( {w,\varepsilon } \right) = \frac{1}{2}\ EW{\varepsilon ^2} + 2{\Gamma _e}\varepsilon + {E_e}{\varepsilon ^2} + {{\cal F}_0}, \end{equation*}

where ${\Gamma _e}$ denotes the edge stress, ${E_e}$ is the elastic moduli of the edges and ${{\cal F}_0}$ is the reference energy state. To understand the edge-stress-driven warping in graphene nanoribbons, Shenoy *et al.* [91] considered a 2D half-space, $- \infty < {x_1} < + \infty $ and $0 \le {x_2} < + \infty $. The authors used the perturbation strategy by assuming the out-of-plane deflection $z$ is in the form of

(18) \begin{equation*}z\ = \ Asin\left( {k{x_1}} \right){e^{ - {x_2}/l}}, \end{equation*}

where $k$ is the wave number and the characteristic wave penetration depth is $l$. In the edge (${x_2} = 0$), the periodic rippling size is $\lambda = 2\pi /k$ and the amplitude of the ripples is $A$. The strain components of the GNR could hence be given as [98]

(19) \begin{equation*} {\epsilon _{ij}} = \frac{1}{2}\ \left( {\frac{{\partial {u_i}}}{{\partial {x_j}}} + \frac{{\partial {u_j}}}{{\partial {x_i}}}} \right) + \frac{1}{2}\frac{{\partial z}}{{\partial {x_i}}}\frac{{\partial z}}{{\partial {x_j}}}.\end{equation*}

It is noted that the first term of the right-hand side in Equation (19) comes from the in-plane displacement $( {{u_1}, {u_2}} )$ and this part is usually neglected due to its relatively small contribution to the overall deformation and the strain energy. For compressive edge stresses, warping in the edges is a favorable configuration with lower free energy. The free-energy density associated with different deformation modes, as seen in Equation (17), could then be deduced. The wave penetration depth is then deduced by using the energy-minimization criterion and is related to $\lambda $ as $l\ = 0.23\lambda $ and the most probable amplitude of edge ripples is [91]

(20) \begin{equation*}A\ = {\left[ {\frac{{ - \lambda {\Gamma _e}}}{{\left( {\pi \sqrt {20 + 14\sqrt 7 } } \right)\frac{E}{{18\left( {1 - {\nu ^2}} \right)}} + \frac{{3{\pi ^2}}}{{2\lambda }}{E_e}}}} \right]^{0.5}}.\end{equation*}

Indeed, molecular dynamics simulations with the Adaptive Intermolecular Reactive Empirical Bond Order (AIREBO) potential [99] as implemented in the software package LAMMPS [100] have been used to predict the compressive edge stresses in GNR. The values are 10.5 and 20.5 eV nm^–1^ in the armchair and zigzag edges, respectively. The predicted penetration depth and rippling amplitude are in good agreement with the corresponding results from MD simulations, as shown in Fig. 2b–d. A combination of edge stress and the low bending resistance of GNR could be utilized to make complex folding structures, as demonstrated in Fig. 2e by Kim *et al.* [90]. Figure 2f–h shows cutting-edge experiments to monitor mechanical vibrations in suspended graphene sheets [101].

It is worth pointing out that several important factors were not considered in the theoretical analysis summarized in Equations (17)–(19). (i) In reality, a GNR is of finite width. The finite width, in particular when a GNR is only several nanometers in width, could not be captured by the analysis based on semi-infinite 2D space. As revealed by Lu and Huang [102], for graphene nanoribbons of width less than the intrinsic wavelength, the interaction between the two free edges becomes significant, leading to anti-phase correlation of the buckling waves. (ii) Temperature is found to play an important role in the rippling of graphene, which is called thermal undulation. The edge stress is also a function of temperature. (iii) The competition of rippling by thermal undulation with edge stress-driven rippling would lead to some more interesting phenomena. Dewapriya and coauthors revealed that temperature, in combination with free edges, influences significantly the mechanical properties of graphene [103].

### Strength and fracture of pristine graphene

We discuss in previous sections the elastic response of graphene. Beyond the elastic limit, pristine graphene breaks in response to further tension. The stress at which graphene is torn apart characterizes the strength of graphene and is another extraordinary property of this class of amazing 2D materials.

#### Strength

Given the same chemical nature, the strength of single-layer graphene is expected to be the same as that of carbon nanotubes if the edge effect is neglected, as confirmed by the very first experimental measurement of the strength of graphene. Lee *et al.* [7] measured the intrinsic strength of monolayer graphene by nano-indentation in an atomic force microscope. While it is not a standard tensile test to characterize the stress–strain response of materials, the authors extracted the stress–strain curve from the force-depth curve obtained from the indention tests. The inferred Young's modulus of *E* = 1.0 TPa and intrinsic strength of 130 GPa for monolayer graphene match reasonably well with the strength of single-wall carbon nanotubes [10,104,105]. These experiments established the foundation that graphene is the strongest material ever measured. The measured strength of 130 GPa for graphene is about 20% higher than the calculated value by the DFPT at 0 K.

The inferred strain in the graphene sheet directly beneath the diamond indenter at the measured failure load is anomalously large compared to the fracture strains predicted by both the soft-mode and the acoustic analyses. Liu *et al.* [52] calculated the phonon spectra of graphene as a function of uniaxial tension by the DFPT to assess the first occurrence of phonon instability on the strain path, which controls the strength of a defect-free crystal at 0 K. The failure strength to break the zigzag plane (loading along the armchair direction) and the armchair plane (loading along the zigzag direction) are 110 and 121 GPa, respectively. This discrepancy, as further elaborated on by [106], may originate from the strain-shielding effect initiated by mechanochemical interactions at the graphene–indenter interface. Transmission electron micrographs and a molecular model of the diamond indenter's tip together suggest that the tip surface contains facets comprising crystallographic {111} and {100} planes.

A more systematic study on the ideal tensile strength of pristine graphene as a function of the loading orientation by using the DFT and energy-minimization calculations was given by Yin *et al.* [58]. The detailed computational procedure has been described in [107]. By defining $\theta $ as the angle between the tensile loading direction (along the *x*-axis) and the zigzag edge and ${\sigma _b}$ the strength of an individual C–C bond, the critical stresses to break the zigzag plane is defined as

(21a) \begin{equation*} {\sigma _c} = \frac{{{\sigma _b}}}{{\cos \left( {60^\circ + \theta } \right)}}.\end{equation*}

Similarly, the critical stresses to break the armchair plane are

(21b) \begin{equation*} {\sigma _c} = \frac{{2{\sigma _b}}}{{\cos \theta [\cos \left( {30^\circ + \theta } \right) + \cos \left( {30^\circ - \theta } \right)]}}.\end{equation*}

The required stress to break the zigzag plane and that to break the armchair plane at different angle *θ* differ significantly, with the latter being larger than the former. From the energy perspective, however, the zigzag plane should be harder to break, as it has greater edge energy than that of the armchair plane [108]. This difference implies that one may need to distinguish the brittle fracture governed by the ideal strength from that by the edge energy.

#### Fracture

In the presence of cracks, graphene sheets typically break along the initial crack. A comprehensive review on the fracture behavior of graphene has been given by Teng *et al.* [28]. As there is no perceivable macroscopic plastic deformation in the stress–strain response of a graphene, graphene is believed to be brittle in nature—that is, single-layer graphene fractures at the nominal peak stress. It exhibits negligible plasticity until its failure at room temperature [8]. As discussed in the ‘2D long-range crystalline order’ section above, its elastic response is isotropic at small strain. Here we focus on the directionality of fracture in graphene and its fracture toughness measurement.

According to the Griffith criterion, the critical fracture stress ${\sigma _f}$ beyond which a pre-cracked isotropic stripe under mode I loading would extend is given as [109]

(22a) \begin{equation*} {\sigma _f} = \frac{1}{{F\left( \phi \right)}}\ \sqrt {\frac{{E\Gamma }}{{{\rm{\pi }}a}}} , \end{equation*}

where *E* is Young's modulus and $\Gamma $ is the apparent fracture resistance of the crack plane. In brittle materials, $\Gamma $ is regarded as the surface energy for 3D materials and the edge energy for 2D materials. In Equation (22), $F( \phi )$ is a geometrical factor given by [110]

(22b) \begin{eqnarray*} F\ \left( \phi \right) &=& \left( {1 - 0.025{\phi ^2} + 0.06{\phi ^4}} \right)\sqrt {\sec \left( {\frac{{{\rm{\pi }}\phi }}{2}} \right)}\nonumber\\ &&{\rm{and\ }}\phi \ = \frac{W}{{2a}},\!\!\!\!\!\!\! \end{eqnarray*}

where $W$ is the width of the stripe with a central crack of length 2*a*.

Both $\Gamma $ and the critical crack size are of practical importance. Zhang *et al.* [111] measured the fracture toughness using a nanomechanical device in a scanning electron microscope. The fracture toughness of graphene was measured by obtaining the critical stress intensity factor of pre-cracked graphene and then deducing its critical strain energy release rate to be $\Gamma = \ 15.9\ {\rm{J}}{{\rm{m}}^{-2}}$. In contrast, the measured fracture toughness of the CVD-grown graphene by Hwangbo *et al.* [112] is about ${K_C} = 10.7 \sim 14{\rm{MPa}} {\sqrt m }$) and this number is higher than that from Zhang *et al.* [111]. Note that the two groups used different testing techniques: Zhang *et al.* [111] tested free-standing graphene using a micromechanical system while Hwangbo *et al.* [112] employed a pressure-bulge testing setup. Whether the difference came from the geometrical effects (e.g. possible rippling in the graphene during bulge tests will enlarge the apparent fracture resistance) remains to be explored with well-controlled experiments. Via comparative *in-situ* fracture toughness testing on single-edge V- and U-notched multi-layer graphenes and boronitrenes BN in a high-resolution transmission electron microscope (HRTEM), Wei *et al.* [113] reported the fracture toughness of multi-layer graphene and boronitrene to be ${K_{IC}} = 12 \pm 3.9$ and ${K_C} = 5.5 \pm 0.7{\rm{\ MPa}}\sqrt m $, respectively.

More recently, numerical simulations have shown that the fracture behavior in graphene is orientation-dependent [114]. Table 1 gives the fracture resistance of graphene when the initial crack is along different chirality. Here the orientation of single-layer graphene is described by two vectors ${{\boldsymbol{a}}_1}$ and ${{\boldsymbol{a}}_2}$, and the crack edge is described by a chiral vector ${{\boldsymbol{C}}_h} = n{{\boldsymbol{a}}_1} + m{{\boldsymbol{a}}_2}$. The armchair and the zigzag directions correspond to ${{\boldsymbol{C}}_h} = ( {1,1} )$ and ${{\boldsymbol{C}}_h} = ( {1,0} )$, respectively. It is seen that the difference in *Γ*_G_ (predicted by the Griffith criterion) or Γ_MD_ (from direct MD simulations) along different orientations is rather small. However, a crack prefers to extend along the zigzag edge in graphene, which is due to the local strength-based failure rather than energy-based Griffith criterion [58].

**Table 1.** Crack/loading angle, crack chirality and the apparent fracture resistance ${\Gamma _G}$ predicted by the Griffith criterion (obtained through fitting the strength versus crack length curve by using Equation (22)) and ${\Gamma _{MD}}$ from MD simulations by calculating the energy of free edges along those particular chiralities.

Crack angle *θ*	0^o^	7.5^o^	15.9^o^	22.5^o^	30^o^
Chirality ${{\boldsymbol{C}}_h}$(m,n)	(1,1)	(5,8)	(2,5)	(2,11)	(1,0)
${\Gamma _G}$	15.9	15.1	14.0	13.5	11.0
${\Gamma _{MD}}$	11.7	12.7	13.1	13.4	11.0

## DEFECTS IN GRAPHENE AND MECHANICS OF DEFECTED GRAPHENE

**Figure 3.**
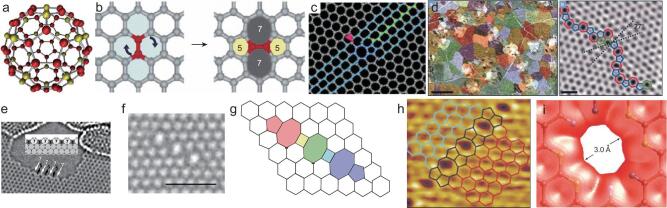
A variety of defects in single-layer carbon allotropes. (a) Pentagons in a fullerene. (b) Stone-Thrower-Wales defect. Adapted from [122], with permission of Elsevier. (c) Observation of 5–7 defects. Adapted from [123], with permission of Springer Nature. (d) GBs in polycrystalline graphene. Adapted from [24], with permission of Springer Nature. (e) Pentagon–heptagon rings in the edge. Adapted from [80], with permission of American Association for the Advancement of Science. (f) and (g) 5–8 defects. Adapted from [126], with permission of American Physical Society. (f) The HRTEM image of 5–8 defects. Scale bar is 1 nm. (g) DFT-optimized structure of the defect for comparison with (f). (h) STM image of a linear defect (chain of two 5-rings with one 8-ring) formed in graphene epitaxial layer on Ni (111) substrate. Adapted from [127], with permission of Springer Nature. (i) Graphene-based membranes with artificial vacancies for molecular separation. Adapted from [128], with permission of American Chemical Society Publications.

It is generally known that many properties of materials and structures are strongly affected or even determined by either intrinsic or extrinsic defects or their combination. This is more evident in one-atom-thick graphene and its derivatives such as carbon nanotubes, fullerenes and so on. Indeed, it seems unavoidable for the presence of defects from geometrical necessity [115]. A typical fullerene, for example, is composed of 12 pentagons (see Fig. 3a). Understanding the mechanical properties of defects is a key challenge but also of particular importance for graphene. In this section, we summarize commonly seen defects in graphene and their mechanical description from up-to-date literature.

### Typical defects

Defects in graphene may be categorized into three types if we differentiate how they are usually produced: thermally dynamically resultant, deformation-introduced and artificial ones. The thermally activated defects are generally of low energy level, such as point vacancy and 5-7-7-5 rings, and 5-8-5 rings. Those defects are seen in graphene fabricated by different methods [116] and they also exist in other carbon allotropes such as fullerene, carbon nanotube and graphite. In particular, in GBs of polycrystalline graphene, such defects are a geometrical necessity to accommodate the incoherent lattice structure at lower energy. However, so far, there have been no reports regarding how frequently a typical thermal dynamic defect may appear. Identifying such defects like vacancy and non-hexagonal rings requires high-resolution tools, which renders a statistic analysis very time-consuming and costly. External deformation could also introduce such kinds of defects; and it may even result in nanoscale cracks and nanoscale pores. Those mechanically triggered defects could be either transient or permanent in response to the removal of the deformation. Furthermore, to achieve a specific functionality, many groups have employed chemical strategies and high-energy bombing methods such as irradiation to generate defects as well. We now introduce in detail those commonly seen defects and explain how they affect the nanomechanics of graphene.

The simplest example of a topological disorder in graphene and other sp^2^-hybridized carbon systems is the Stone-Thrower-Wales defect, usually called Stone-Wales, which results from rotating a C–C bond by 90° with regard to the midpoint of the bond—referred to as the SW transformation—so that four hexagons are turned into two pentagons and two heptagons [117,118] (see Fig. 3b, c). Several derivatives had been identified, including the so-called inverse SW defect [119–122] and the di-vacancy defect [123], which is formed by the removal of two adjacent carbon atoms (Fig. 3i). It has been seen that GBs are dominantly composed of pentagon–heptagon pairs to accommodate the lattice mismatch of pristine graphene flakes at different orientations, which is an important way to realize large-area but polycrystalline graphene, as seen in Fig. 3d. Reconstructed zigzag edges in GNR are also composed of pentagon–heptagon pairs, as proved by Jia *et al.* [80] (Fig. 3e). More recently, Kim *et al.* [124] demonstrated reversible and extended pentagon–heptagon (5–7) reconstruction at zigzag edges, and explored experimentally and theoretically the dynamics of the transitions between the edge configuration states.

By utilizing cutting-edge techniques, Warner *et al.* [125] determined the atomic structure, including the bond length and charge density variations of edge defects within extended arm chair defects in graphene, as well as bond elongation within a pair of 5-8-5 di-vacancies. Irradiation-induced 5-8-5 defects [126] and GBs formed by a group of 5-8-5 defects [127] had also been observed, as shown in Fig. 3f–h, respectively. There is also growing interest in making nanoscale defects in graphene for special applications, such as nanopores (Fig. 3i) in graphene-based membranes for molecular separation [128].

### Pentagon–heptagon ring

**Figure 4.**
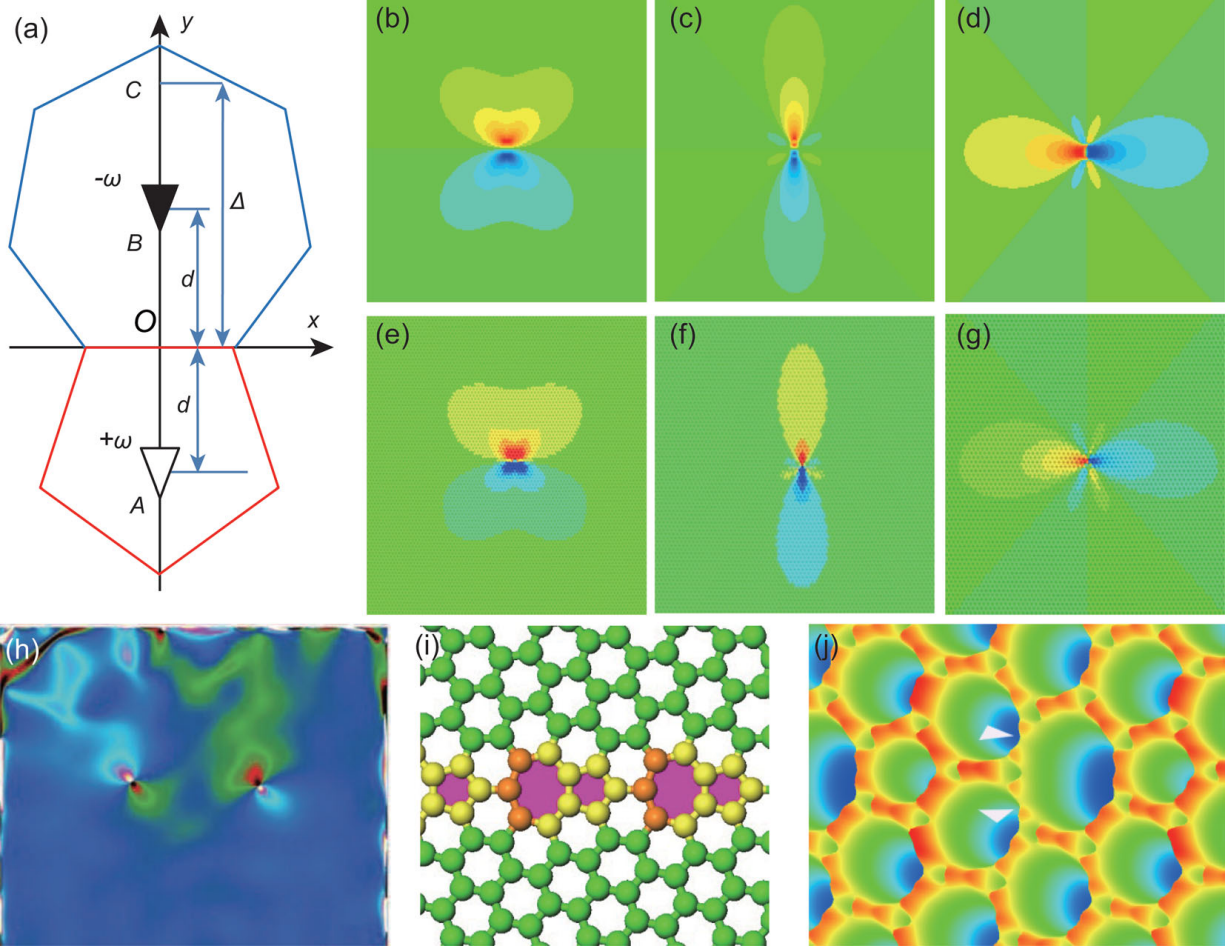
Stress field induced by a 5–7 defect. (a) The structure of a 5–7 ring and its equivalence to a disclination dipole. (b)–(d) The stress contours of *σ*_xx_, *σ*_yy_ and *σ*_xy_ predicted by the disclination dipole model; and (e)–(g) the corresponding contours for *σ*_xx_, *σ*_yy_ and *σ*_xy_ calculated using MD simulations. (h) Experimentally measured shear strain introduced by a 5–7 ring. Adapted from [133], with permission of American Association for the Advancement of Science. (i) and (j) The structure used for DFT calculation and the local weakest bond (shared by the 6–7 ring) in the 5–7 defect. Adapted from [27], with permission of Elsevier.

Among all those different types of defects, the most commonly seen pentagon–heptagon pairs deserve further consideration. Pentagon–heptagon pairs are analogous to dislocations in bulk crystalline materials and are the most important defects in a 2D hexagonal lattice. From a geometrical perspective, a pentagon–heptagon pair resembles a disclination dipole [129–132], which consists of two disclinations of opposite signs. In view of this, Wei *et al.* [26] constructed the stress field of a pentagon–heptagon pair by using the disclination dipole model. The stress components induced by a disclination dipole with a positive disclination residing at (0, –*d*) and a negative one at (0, *d*) (see Fig. 4a) are given as

(23a) \begin{eqnarray*} \frac{{{\sigma _{xx}}}}{{{\sigma _0}}} &=& \frac{1}{2}{\rm{\ ln}}\frac{{{x^2} + {{\left( {y + d} \right)}^2}}}{{{x^2} + {{\left( {y - d} \right)}^2}}} + \frac{{{x^2}}}{{{x^2} + {{\left( {y - d} \right)}^2}}}\nonumber\\ && -\, \frac{{{x^2}}}{{{x^2} + {{\left( {y + d} \right)}^2}}}, \end{eqnarray*}

(23b) \begin{eqnarray*} \frac{{{\sigma _{yy}}}}{{{\sigma _0}}} &=& \frac{1}{2}{\rm{\ ln}}\frac{{{x^2} + {{\left( {y + d} \right)}^2}}}{{{x^2} + {{\left( {y - d} \right)}^2}}} + \frac{{{x^2}}}{{{x^2} + {{\left( {y + d} \right)}^2}}}\nonumber\\ && -\, \frac{{{x^2}}}{{{x^2} + {{\left( {y - d} \right)}^2}}}, \end{eqnarray*}

(23c) \begin{eqnarray*} \frac{{{\tau _{xy}}}}{{{\sigma _0}}} = \frac{{x\left( {y + d} \right)}}{{{x^2} + {{\left( {y + d} \right)}^2}}} - \frac{{x\left( {y - d} \right)}}{{{x^2} + {{\left( {y - d} \right)}^2}}}.\qquad \end{eqnarray*}

The stress contours of ${\sigma _{xx}}$, ${\sigma _{yy}}$ and ${\tau _{xy}}$ predicted by the disclination dipole model, as seen in Fig. 4b–d, agree well with the large-scale MD simulations (Fig. 4e–g) produced by a pentagon–heptagon pair using a 1000 × 1000-nm graphene sample in a vacuum. Warner *et al.* [133] measured the strain fields of individual pentagon–heptagon pairs and showed how the defect deforms graphene by elongation and compression of C–C bonds, shear and lattice rotations. The experimental result shown in Fig. 4h agrees well with the theoretical prediction. The stress fields described by Equation (23) significantly differ from those of a dislocation within the core, although they are similar for the region far away from the core. The accumulated effect by the local difference is important for understanding grain-boundary strength, as explained below.

### Grain boundary

With the stress fields of individual pentagon–heptagon pairs known, in particular the weak bond associated with the defect (see Fig. 4j and k), it is important to understand its influence on the strength of graphene. Accompanied with the development of synthesis techniques capable of generating large-area graphene, GBs were found to exist in most as-fabricated graphene [134–138] and those GBs are composed of pentagon–heptagon defects. Indeed, understanding how GBs primarily composed of pentagon–heptagon defects in graphene alter its physical properties is of both scientific and technological importance [24,25,138–143]. Recently, Biro and Lambin [144] reviewed the literature on GBs in graphene, with a focus on the experimental findings on graphene grown by CVD under a very wide range of experimental conditions (temperature, pressure hydrogen/hydrocarbon ratio, gas flow velocity and substrates for growth). Here we summarize the theoretical advances on constructing the grain-boundary and mechanical-strength relationship.

#### The formation of 2D GBs

**Figure 5.**
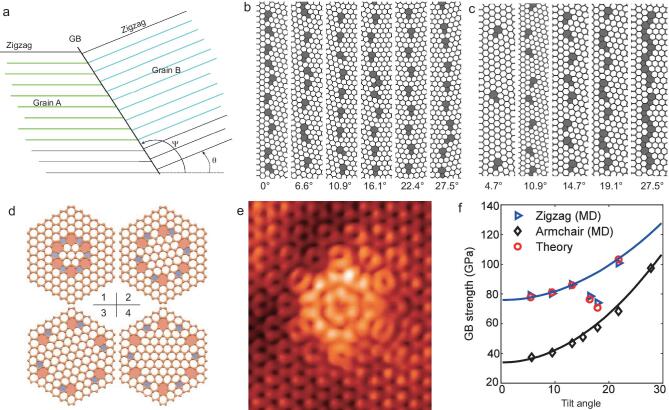
Grain-boundary structure in a graphene sheet. (a) The two degrees of freedom in a GB in 2D materials—the mis-orientation *θ* and the GB rotation *ψ*. (b) Atomic structures of GBs with constant *θ* but different *ψ* from 0 to 27.5^o^. (c) GB structures with constant *ψ* but *θ* varying from 4.7 to 27.5^o^. Adapted from [27], with permission of Elsevier. Note GB defect density depends on *θ* but is insensitive to *ψ.* (d) and (e) Topographic images of rotational GBs. Adapted from [142], with permission of American Physical Society. (d) Four rotational GBs of different sizes (1 to 4) and (e) STM observation of type 1 GB in the epitaxial graphene on SiC. (f) The relationship of the strength versus tilt angle (mis-orientation) of symmetrical GBs. Adapted from [26], with permission of Springer Nature.

Geometrically, a general GB in 3D polycrystalline materials is characterized by five degrees of freedom: three from the relative rotation of adjacent grains and two due to the angular directions of the planar GB. For 2D polycrystalline graphene, only two rotational degrees of freedom are needed to define a general GB: the mis-orientation of the two grains ($\theta $) and the rotation of the boundary line itself ($\psi $), as shown in Fig. 5a. For here and in what follows, we refer to $\theta $ as grain mis-orientation and $\psi $ as GB rotation. Figure 5b shows the atomic structures of GBs with constant *θ* but different *ψ* from 0 to 27.5^o^. The GB structures with constant *ψ* but *θ* varying from 4.7 to 27.5^o^ are shown in Fig. 5c [27]. It is interesting to note that the density of GB defects depends strongly on grain mis-orientation but is not sensitive to GB rotation.

The kinetic mechanisms leading to the type of GBs have been broadly investigated. Cockayne *et al.* [142] presented a class of topological defects in graphene, composed of a rotating sequence of dislocations that close to themselves, forming grain-boundary loops that either conserve the number of atoms in the hexagonal lattice or accommodate vacancy or interstitial reconstruction, while leaving no unsatisfied bonds. Such a grain-boundary loop resembles a ‘flower’ pattern in scanning tunneling microscopy (STM) studies of epitaxial graphene grown on SiC(0001), as shown in Fig. 5d and e.

Theoretically, Seymour and Provatas [145] developed a new structural phase-field crystal (PFC) model that allows the stabilization of graphene, as well as its coexistence with a disordered phase. The model is adopted for efficient simulations for the nucleation and growth process of polycrystalline 2D materials, which shows the defect structures produced in CVD-grown polycrystalline graphene. Ophus *et al.* [146] then characterized the structure of many different GBs in single-layer graphene using HRTEM, and introduced a new algorithm for generating grain-boundary structures for a class of hexagonal 2D materials. The authors found excellent agreement between the simulated and experimentally observed GBs.

#### Strength of an individual GB

The formation of GBs would affect the strength of materials. The theoretical efforts in studying the strength of GBs began with the exploration by Grantab *et al.* [25], who found that more GB defects could counter-intuitively give rise to higher strength in tilt GBs. A more comprehensive analysis by Wei *et al.* [26] demonstrated that GB strength can either increase or decrease with the tilt, and the behavior can be well explained by the stress fields introduced by pentagon–heptagon pairs as shown in the ‘Pentagon–heptagon ring’ section above. It is not just the density of defects that affects the mechanical properties, but also the detailed arrangement of defects is important. Well-stitched high-angle GBs in graphene would only slightly degrade its strength.

In the special case that GBs are symmetrical tilt ones, the strengths of tilt GBs increase as the square of tilt angles if pentagon–heptagon defects are evenly spaced [26]. In that scenario, the residual stress ${S_{xx}}$ in an infinitely long tilt GB with tilt angle *θ* (or equivalently GB mis-orientation) is given as

(24) \begin{equation*} \frac{{{S_{xx}}}}{{{\sigma _0}}} = - \frac{{2{\pi ^2}\Delta d}}{{3h_d^2}}\frac{{{\theta ^2}}}{{{\omega ^2}}}, \end{equation*}

where $\Delta $, $d$ and ${h_d}$ are geometrical parameters associated with the defects and their arrangement, $\omega $ is the rotational strength of the disclinations and ${\sigma _0} = E\omega /4\pi $, where $E$ is the Young's modulus. The negative sign in Equation (24) suggests that the residual stress introduced by the neighboring defects in the GB is compressive, which could compensate for the exerted tensile stress and lead to high strength in the absence of interaction from other defects. Notably, the compressive stress at positive $\Delta $ (above the origin, on the side of the negative disclination) is proportional to ${\theta ^2}$. It is also important to know that $\omega $ is not $\pi /3$ from simple geometrical consideration, but a value depending on the type of GB. Table 2 lists the corresponding geometrical and material parameters for both armchair and zigzag tilt GBs [26]. An excellent agreement between theoretical analysis and molecular dynamics simulations was found, as shown in Fig. 5f.

**Table 2.** The geometrical and material parameters used for Equation (24) to obtain the theoretical curves shown in Fig. 6f for both armchair and zigzag tilt GBs.

Name units	$\omega $ (degree)	$\Delta $ (*a*)	${h_d}$ (*a*)	$d$ (*a*)	${\sigma _{y0}}$ (GPa)
Zigzag	21.8	1.5	4.7	0.8	76
Armchair	27.8	3.2	6.3	1.5	33

In parallel to the theoretical analysis, two groups reported the strengths of individual GBs experimentally. Lee *et al.* [8] combined the structural characterization by means of transmission electron microscopy with nano-indentation tests to study the mechanical properties of CVD-grown graphene films with different grain sizes. They found that the strength of graphene films is only slightly reduced despite the existence of GBs. Indentation tests directly on GBs confirmed that they are almost as strong as the pristine ones. Rasool *et al.* [9] measured the strengths of suspended bi-crystal graphene membranes with different GB mis-orientations, and revealed that GBs with large mis-orientations in polycrystalline specimens have higher strengths than their low-angle counterparts. These high-angle GBs even show strength comparable to that of single-crystal graphene. The higher strength in GBs of larger mis-orientations could be well explained by the larger compressive residual stress introduced by more neighboring pentagon–heptagon defects.

#### Strength of polycrystalline graphene

The understanding on the strength of individual GBs paves the road for understanding the strength of polycrystalline graphene containing abundant GBs, which is of significance in engineering practice. Suk *et al.* [147] studied the failure of CVD-grown polycrystalline graphene by nano-indentation testing in a scanning electron microscope. Their measurement indicates that graphene membranes without any GBs had a failure strength of 45.4 ± 10.4 GPa, compared to 16.4 ± 5.1 GPa for those with GBs. A large variation in strength is seen. Shekhawat and Ritchie [148] investigated the statistical fluctuations in the toughness and the strength of polycrystalline graphene containing inherent nanoscale line and point defects—GBs and grain-boundary triple junctions. They showed that the statistical variation in the toughness and the strength can be understood with ‘weakest-link’ statistics, and elucidated the origins of the grain-size dependence of its strength and toughness. Such ‘weakest-link’ statistics may be employed to explain the controversial observations from MD simulations. Sha *et al.* [149] showed that the breaking strength and average grain size in graphene follow an inverse pseudo Hall–Petch relation, in agreement with experimental measurements [9]. They also explained this inverse pseudo Hall–Petch relation by reasoning that the weakest-link determines the failure behavior of brittle materials. On the other hand, Song *et al.* [150] reported a pseudo Hall–Petch strength reduction in polycrystalline graphene where samples of smaller grains exhibit higher strength. The observed crack localization and strength behavior were interpreted by a dislocation-piled-up model. The insensitiveness of flaws in nanocrystalline graphene could be originated from the statistical nature of defective GBs in competition with pre-existing flaws [151]. An interesting observation was made by Lin *et al.* [152] on the fracture behavior of the two-layer stacked graphene membranes using nano-indentation performed with atomic force microscopy. The authors observed distinctly different fracture force distribution of stacked graphene from that of monolayer graphene: the stacked graphene membrane becomes less sensitive to the defects during nano-indentation, improving the overall performance of the graphene membranes.

It is worth noting that the original Hall–Petch relation is only strictly applicable to crystalline metals in which their strength is governed by the dislocation activities. It describes the strengthening mechanism resulting from GBs by retarding dislocation motion; smaller grains in bulk polycrystalline materials imply more GBs to serve as barriers to dislocation motion. Considering that no plastic deformation carrier is found in graphene at room temperature, applying the Hall–Petch relation in graphene might be a wrong shot. The ‘weakest-link’ statistics could be better suited to describe the grain-size-dependent strength in graphene. The strength–grain-size relationship is all governed by the coherence of GBs and triple junctions in graphene.

### Defect engineering

As every coin has two sides, defects in graphene could be engineered to realize particular structure configuration or certain exciting properties. Orlikowski [153] discussed the possibility of employing a combination of ad-dimers and strain to form nanotube-based quantum dots. By using the DFT calculations, Lust and Carr [154] reported a set of stable domain structures including blisters, ridges, ribbons and meta-crystals. Through such a treatment, graphene with some particular chemical properties or ultra-fine pore could be also realized, while the latter could be utilized for specialized filtering and selective membranes for chemical and biological applications [155,156].

For free-standing graphene, defects may introduce local compression given the graphene is flat. The compressive state is unstable, which leads to out-of-plane bulging from which the local high stress is relaxed by warping [10,26,157,158]. Such mechanical deformation may be described by the generalized Föppl-Von Karman equation for a flexible solid membrane [70] and be utilized to design topological surfaces [70,71,159,160], given that patterned defects could be synthesized.

Instead of changing graphene layers, Wang and Crespi [161] explored a way to engineer GBs in 2D crystals by controlling the substrate. They demonstrated that depositing graphene on a substrate of non-zero Gaussian curvature may facilitate the growth of *finite-length* GBs that terminate abruptly within a mono-crystalline domain. By properly designing the substrate topography, these GBs can be placed at desired locations and at specified misfit angles. New properties specific to certain grain-boundary geometries, including magnetism and metallicity, can thus be engineered into 2D crystals through topographic design of their growth substrates.

### Defect motion

The pentagon–heptagon defects–dislocations in the hexagonal 2D lattice have been investigated for motion possibility with the aim to make the strongest material to deform plastically.

**Figure 6.**
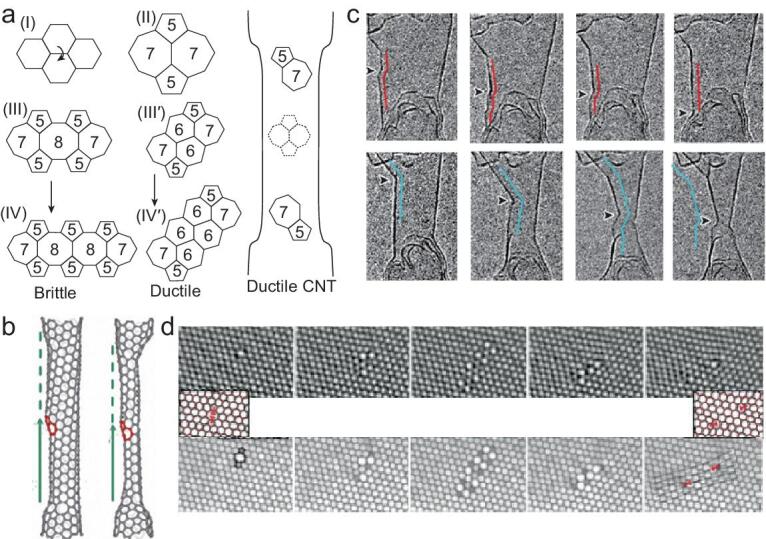
Motion of defects in single-layer carbon allotrope. (a) Illustration to show the ductile versus brittle deformation mechanisms by the motion of 5–7 rings, (II) to (IV) for brittle and (II) to (III’) and (IV’) for ductile deformation. Adapted from [162], with permission of American Institute of Physics Publishing. (b) Molecular dynamics simulations to show plastic deformation mechanism in CNTs. Adapted from [163], with permission of American Physical Society. (c) *In-situ* observation of kink motion in a SWCNT under tension. Adapted from [165], with permission of American Physical Society. Sketches in the figures show the change in the shape and position of the kinks. (d) *In-situ* observation of defect formation, transformation and separation of a single-layer graphene. Adapted from [168], with permission of Springer Nature.

The Stone-Wales defect has received a considerable amount of attention, as it has the lowest formation energy among all intrinsic defects in the graphene system. As proposed by Yakobson [162], a pentagon–heptagon defect presumably plays an important role in plastic deformation of carbon nanotubes (CNTs) under tension by accommodating the strain, as detailed in Fig. 6a. Molecular dynamics simulations by Ding *et al.* [163] recaptured the deformation scenarios, as seen in Fig. 6b. Such simulations could also help to shed light on the diffusion, coalescence and reconstruction of vacancy defects in graphene layers at elevated temperatures [164]. The suggested plastic deformation and kink formation in CNTs seem to agree well with the experimental observation [164]. Huang *et al.* reported that kink motion, reminiscent of dislocation motion in crystalline materials, contributed to plastic deformation in all carbon nanotubes when being tensile loaded at high temperatures [165]. The deformation mechanism proposed by Yakobson [162] describes the main route of mechanical relaxation in a series of 2D nano-crystals, suggesting a brittle cleavage at low temperatures, but plastic flow is likely at high temperatures or under electron radiation [166]. In both cases, deformation starts with diatomic rotation, which produces a dislocation dipole with the pentagon–heptagon cores. Under high stress, the defects depart from each other, leaving behind a permanent shearing [162,167–169].

HRTEM have been widely employed to exploit the motion of defects in graphene. Warner *et al.* [133] reported the stepwise dislocation movement along the zigzag lattice direction mediated either by a single bond rotation or through the loss of two carbon atoms. The strain fields were determined, showing how the dislocations deform graphene by elongation and compression of C–C bonds, shear and lattice rotations. A cutting-edge aberration-corrected TEM study by Lehtinen *et al.* [168] demonstrated how the impinging energetic electrons stimulate atomic-scale morphological changes in graphene. The full life cycle of transformations from birth to annihilation was seen *in situ* and atom by atom (see Fig. 6d). Also with an aberration-corrected TEM, Kurasch *et al.* [169] used the energy of imaging electrons to stimulate individual bond rotations in the GB core region. They then observed *in-situ* atom-by-atom GB migration and its dependence on GB curvature. STM was also broadly used to examine the growth of graphene on the Si-terminated facet of 6H-SiC (0001) [170]. The initial stages of ultrahigh vacuum graphitization resulted in the growth of individual graphene sheets on SiC terraces. The authors demonstrated that multi-layer thickness resulted in a high density of defects, located predominantly below the first layer of graphene.

The aforementioned research advances our understanding on the mobility of defects in graphene. The plastic deformations revealed so far, however, are contingent upon the assistance of the impinging energetic electrons or the help of high temperatures. At room temperature, graphene sheets, either single-layer or multiple-layer, is brittle in nature. Even at high temperatures or under irradiation, there is no literature reporting perceivable plasticity in graphene. While the mechanical performance of graphene at high temperatures is very appealing, there seems to be a long way to go before achieving macroscopic plasticity in graphene.

## GRAPHENE ON A SUBSTRATE

**Figure 7.**
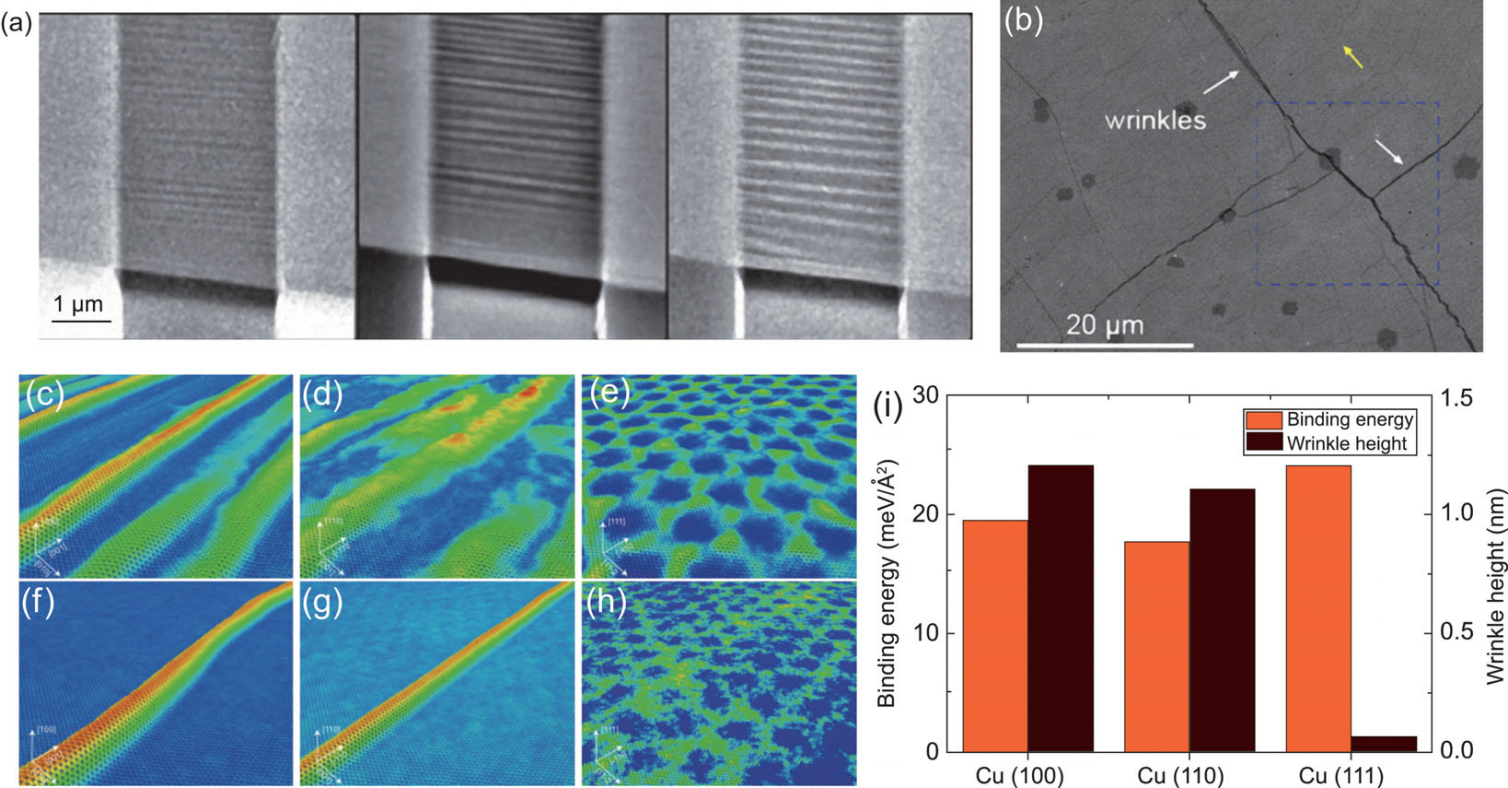
Wrinkling of graphene on a substrate (experiments and simulations). (a) Scanning electron microscopy (SEM) images of a membrane before annealing (left), after annealing to 425 K (middle) and to 475 K (right). Notice the increase in wavelength and amplitude of the ripples with the annealing temperature. Adapted from [183], with permission of Springer Nature. (b) SEM image of graphene with wrinkles on SiO_2_/Si substrate. Adapted from [181], with permission of American Chemical Society Publications. (c)–(h) Morphology of graphene on Cu substrate of different crystallographic planes. (c)–(e) Morphology at 300 K after initial relaxation, (c) Cu (100), (d) Cu (110), (e) Cu (111). The morphology of graphene on (f) Cu (100), (g) Cu (110), (h) Cu (111) after a sufficiently long period. Color reflects the height of wrinkles. (i) The dependence of binding energy and wrinkle height on Cu crystallographic planes. Adapted from [181], with permission of American Chemical Society Publications.

Monolayer graphene exhibits rich morphology, including rippling in free-standing graphene introduced by stresses (see Fig. 7a) or thermal undulation. The mismatch of the coefficient of thermal expansion (CTE) between graphene and the substrate could induce strong compressive strain ($\varepsilon = {\rm{\Delta }}T( {{\alpha _g} - {\alpha _{Cu}}} )$) for a temperature drop ${\rm{\Delta }}T$ in the graphene film (see Fig. 7b–i). Mechanically, two features are important to describe a graphene layer on a substrate: the adhesion energy and wrinkling. The wrinkling in graphene on a substrate is highly sensitive to the adhesion energy between graphene and the substrate [171,172].

### Adhesion

The adhesion between the graphene layers (in the case of multi-layer graphene [18,173] or that between graphene and the substrate [13,174–178]) is critical for the transfer of the graphene layer as well. A number of experiments have been performed to obtain the adhesion energy of graphene on different substrates [13,175–178]. There are two striking observations that are worth noting: (i) the values of graphene-substrate adhesion energy are greater than those of similar micromechanical systems, and one may attribute this characteristic to the low bending resistance of graphene, which promotes conforming contact of graphene with the substrates of arbitrary topography; and (ii) the adhesion energy between single-layer graphene and a substrate is significantly higher than that of multi-layer graphene on the same substrate. For graphene of more than two layers, the adhesion energy on the same substrate remains nearly the same. For example, the adhesion energy of monolayer graphene and silicon oxide was found to be $0.45$ and $0.31\ J\ {m^{ - 2}}$ for samples containing two to five graphene layers [13,171,172]. The second observation reminds us to exercise caution when discussing the interaction of graphene with substrates.

### Wrinkling

Once the strain energy resulting from thermal mismatch during cooling of the graphene-substrate system is large enough to overcome their adhesion, the graphene layer buckles to form wrinkles, relaxing its in-plane compression at the expense of interfacial energy due to delamination and bending energy in the wrinkles. The wrinkles can form during both the growth and the transfer processes, and are very hard to release.

The wrinkling of graphene could be described using the continuum theory for thin elastic sheets. Cerda and Maha [179] deduced a general theory of wrinkling using elementary geometry and the physics of bending and stretching. Their main results include the scaling laws between the wavelength of the wrinkles $\lambda $ with stiffness $K$ due to an ‘elastic substrate’ effect, with $\lambda \ \sim \ {K^{ - 1/4}}$, and the amplitude of the wrinkle $A\ \sim \lambda $. Given different crystallographic planes have distinct density of atomic packing and surface energy, there is a strong dependence of wrinkling patterns on the substrate on which graphene is grown [180,181].

For simplicity but without the loss of general physics, we may describe the out-of-plane displacement *z* of the wrinkles by a sinusoidal function:

(25a) \begin{equation*}z = Asin\left( {2\pi y/\lambda } \right)\!, \end{equation*}

where $A$ is the amplitude and $\lambda $ the wavelength. Wang and Devel [182] showed that the dependence of the ripple structure on the compressive edge strain $\varepsilon $ should be governed by

(25b) \begin{equation*} {\lambda ^4} = \frac{{4{\pi ^2}\nu {L^2}{t^2}}}{{3\left( {1 - {\nu ^2}} \right)\varepsilon }}.\ \end{equation*}

Bao *et al.* [183] reported the very first direct observation and controlled creation of 1D and 2D periodic ripples in suspended graphene sheets, using both spontaneously and thermally generated strains (see Fig. 7a). They demonstrated that the ripple orientation, wavelength and amplitude are tunable by controlling the boundary conditions and making use of the negative thermal expansion coefficient of graphene. Such manipulation of wrinkles may lead to advanced application of graphene-based nanoelectronics [184]. Under the influence of substrate, Tapasztó *et al.* [185] observed periodic rippling of nanometer-scale wavelength in the suspended graphene membranes under thermal strain using STM. The observed nano-rippling mode differs significantly from the predictions of the continuum mechanics model, which indicates the breakdown of applying the plate theory for graphene.

## PERSPECTIVES

Regardless of the tremendous progress that has been achieved on the deformation behavior of graphene in response to mechanical or thermal undulation, there are still compelling needs on several key issues that cannot be satisfactorily addressed using the existing theories or computational tools. We comment below on three mechanically related issues that are of significance for the morphology manipulation of graphene in contact with substrates or under thermal undulation. Those issues call for further development in theory and computational tools for better predicting the mechanical behavior of graphene.

### Interlayer van der Waals interaction

The stacking of multiple layers of graphene forms graphite. The interlayer bond nature and its effective description are important for multi-layer graphene as well as interactions of graphene with other structures. From a recent perspective by Geim and Grigorieva [186], research on graphene and other 2D atomic crystals made layer by layer in a precisely chosen sequence, often referred to as ‘van der Waals’ heterostructures, may result in unusual properties and new phenomena. Such interaction, not surprisingly, would have a significant influence on the physical properties of those layered 2D structures [187–189]. Van der Waals interaction could also be used for epitaxy, which is often limited by the need for lattice matching between the two material systems. This strict requirement is relaxed with epitaxy on layered 2D materials, which is mediated by weak van der Waals interactions, and also allows facile layer release from 2D surfaces [190]. The mechanical behavior of multiplayer graphene is fully determined by the interlayer van der Waals force. The interlayer shearing and rigidity affect the stiffness of a ‘van der Waals’ heterostructures [173]. The strength and fracture toughness also rely on how monolayers are bonded together.

While the in-plane deformation in graphene could be well captured by the all-electron DFT calculations, popular density functionals for most first-principles-based calculations are unable to describe correctly van der Waals interactions resulting from the dynamical correlations between fluctuating charge distributions [191]. In most atomistic simulations, practitioners chose the empirical Lennard-Jones (L-J) potentials to represent the interlayer interaction. Now the development is moving towards using the empirical correction for dispersion (van der Waals) effects (DFT-D method) by adding a semi-empirical dispersion potential to the conventional Kohn-Sham DFT energy to work around the limitation of the DFT method and give better accuracy in contrast to the L-J approximation [191–193]. Different versions of the correction have then been developed. In a recent study by Grimme *et al.* [194,195], more complicated, geometry-dependent dispersion coefficients and Becke-Jonson damping were taken into account. Cooper *et al.* [196–198] developed an exchange functional that is compatible with the nonlocal Rutgers-Chalmers correlation functional (van der Waals density functional, vdW-DF). This functional, when employed with vdW-DF, demonstrates remarkable improvements on intermolecular separation distances while further improving the accuracy of vdW-DF interaction energies.

The DFT-D method and its derivatives have been broadly employed to capture the physics and mechanics of multi-layer graphene [199–202] and the interaction of graphene with metal substrate [203]. It should be noted that the superlubricity in graphite [204] and weak shear strength [205] in graphene ought to be connected with the vdW characteristics between carbon layers. So far, even the DFT method and its derivatives are semi-empirical. They may work well in capturing one or several physical properties but are not justified to be accurate in general or other properties. A consensus on a reliable interaction formula to capture the vdW interaction in graphene is far from being reached. Another growing field of graphene is the interaction of graphene with other materials where an accurate atomic potential is desired for the commonly known fact that the accuracy of an atomistic simulation is by far no better than the potential one uses.

### Limitation of thermal–mechanical coupling

At high temperatures, ripples are formed in free-standing graphene due to thermal fluctuations. The amplitude of ripples may be approximated from the free energy of the interface by a surface tension $\gamma$. If we consider the crumpled graphene surface, free energy in terms of surface tension times the surface area can be written as [206]

(26) \begin{eqnarray*} \left( \psi \right) &=& \ \gamma \smallint \sqrt {1 + {{\left| {\vec{\nabla }h} \right|}^2}} dxdy \nonumber\\ && \approx\, {\psi _0} + \frac{1}{2}\gamma \smallint {\left| {\vec{\nabla }h} \right|^2}dxdy, \end{eqnarray*}

where $h$ is the height of the graphene at each 2D position $\ \vec{r} = ( {x, y} )$; the height–height correlation function may then be written as

(27a) \begin{eqnarray*} {{( {h( {\vec{r}} ) - h( {\vec{0}} )})^2} =}\nonumber\\ && \frac{{\smallint D(h( {\vec{r}}){{| {h( {\vec{r}}) - h( {\vec{0}})}|}^2}{e^{ - \psi /kT}}}}{{\smallint D(h( {\vec{r}}){e^{ - \psi /kT}}}}. \end{eqnarray*}

The integral of the above equation leads to

(27b) \begin{equation*} {( {h( {\vec{r}} ) - h( {\vec{0}})})^2} \approx \frac{{kT}}{{\pi \gamma }}\ln ( {r/a}), {\rm{as}}\ {\rm{r}} \to \infty , \end{equation*}

where *a* is a microscopic length. Equation (27) suggests that there is a divergent height–height correlation. The large $r$ behavior is the signature of a rough surface at high temperature. In contrast, ${( {h( {\vec{r}} ) - h( {\vec{0}} )} )^2} \approx {\rm{const}}$, as $r \to \infty $ at low temperature, and we might expect a ‘smooth’ interface. How such a transition from smooth to rough surface influences the phononic thermal conductivity of a suspended 2D material remains unclear. Rationales may help to shed light on the increase in thermal conductivity of GNR with the sample size even when the size is beyond tens of microns.

### Scale-up

In its 2D form, graphene is thought to be the strongest of all known materials. It is desirable to use such low-dimensional carbon structures as building blocks to realize 3D engineering materials and structures that may inherit their superb properties. There is much past and ongoing research aiming to utilize the amazing mechanical properties of graphene and other carbon allotropes as strengthening agent in 3D structures [207,208] or composites [209–213].

In reality, the scale-up leads to a substantial degradation of properties that we desire to retain. The realized mechanical and thermal properties of 3D carbon materials, by staggering graphene sheets or vertically grown carbon nanotube arrays, are significantly lower than those of individual graphene sheets or individual CNTs [211]: the strongest graphene paper reported in the literature has a strength 2∼3 orders of magnitude lower than that of graphene [210,212]. The huge gap in the thermal and mechanical properties between the low-dimensional carbon allotropes and their 3D derivatives originates from the dissimilar bonding characteristics between carbon atoms within graphene or CNTs and the architected 3D engineering materials: the intra-structure bonding is covalent in nature, while van der Waals bonding dominates between different layers/tubes or with other materials [212]. Such heterogeneous bonding leads to property inheritance as a mission impossible.

**Figure 8.**
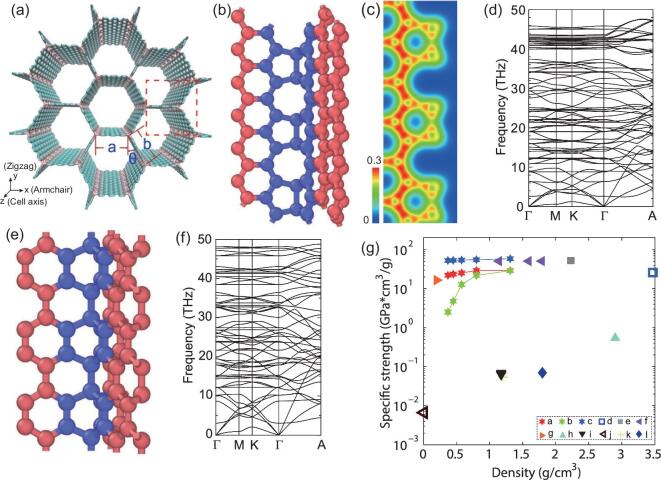
Stable C-honeycomb structure. (a) Atomistic structure of C-honeycomb and the coordinate system defined based on the honeycomb. (b) Local atomistic structure at the zigzag triple junction of a C-honeycomb. (c) The electron density at the junction region. (d) The phonon dispersion of the stable C-honeycomb with cell size of 5.8 Å where all phonons are with positive frequencies. (e) Local atomistic structure at the armchair triple junction of C-honeycomb. (f) The phonon dispersion of the stable C-honeycomb with cell size of 5.2 Å where all phonons are with positive frequencies. (g) Specific strength of C-honeycomb of different cell sizes and other carbon-based materials. Adapted from [215], with permission of American Chemical Society Publications.

The interest in finding 3D carbon structures has lasted for decades. Recent success in the synthesis of carbon honeycomb (C-honeycomb) [214] shows great potential in scaling up the low-dimensional carbon allotropes to 3D engineering materials and structures while retaining strong covalent bonding. Such a C-honeycomb structure may circumvent the change of bonding while using graphene as basic building blocks. Pang *et al.* [215] reported the atomistic structure of a stable 3D C-honeycomb structure. The authors demonstrated that a combination of sp^2^ bonding in the wall and sp^3^ bonding in the triple junction stabilizes the C-honeycomb. The detailed stable structures and their phonon dispersion are given in Fig. 8. Due to the low density in such a stable 3D-architectured C-honeycomb with covalent bonding, its specific strength of C-honeycomb could be the best in structural carbon materials, and its specific thermal conductivity is also much better than most metal and high thermal conductivity semiconductors, as clearly seen in Fig. 8g.

### Summary and outlook

Since the successful peeling of graphene by Novoselov *et al.* [1], the mechanical properties of graphene have attracted great attention from the research community. Examining the applicability of the classical mechanics theories to the materials with extreme aspect ratios of large in-plane dimensions over one-atom-thick limits, revealing new mechanical behaviors and developing new theories to capture the nanomechanics of graphene, has been of paramount interest. In this work, we reviewed the current progress in the in-plane and out-of-plane mechanics of graphene. The structure–mechanical property relationship in graphene, in terms of its elasticity, strength, bending, with or without the defects, is presented. Although derived and validated at the macroscopic scale, several continuum theory formulae have been successfully applied to describe the elasticity of single-layer graphene. It is also suggested that the currently developed theories and modeling tools for the deformation of graphene are probably applicable to other members in the growing family of 2D materials. At the end, we also commented that several mechanics-related issues need to be addressed in the near future for better understanding the fascinating 2D carbon allotrope.

The exploration of the extraordinary properties of graphene is strongly mechanically relevant: the original theory of structural instability in 2D crystals is a pure mechanics concept; the very first way of obtaining single-layer graphene is through mechanical exfoliation by utilizing its ultra-small adhesion. We indeed would expect that mechanics may continue to play a pivotal role for the synthesis and the application of graphene. When graphene is functionalized as a critical component, intrinsic defects, pre-straining and morphology evolution due to lattice mismatch would all affect the final performance of the graphene-based system. For instance, wrinkles in graphene may lead to anisotropic electrical mobility [216], local charge accumulation [217], corrosion-resistance degradation [218] and mechanical-strength and thermal-conductivity reduction [217,219]. In contrast, we may also use graphene as a building block for further development of composite materials and heterostructures. In the latter circumstance, the properties of graphene including strength, fracture toughness and adhesion at the microscopic level will play important roles for the macroscopic properties of the hierarchically architectured materials. In addition, the rich morphology, abundant defects, as well as the defect–defect interaction and morphology–defect coupling give rise to further challenges to obtain the structure–property relationship of graphene-based macroscopic materials. Given the attraction of graphene for nanomechanical systems [220] and composite materials [210,212], many of the mechanical issues that are responsible for reliability and durability need to be addressed before real-world application.

## References

[1] Novoselov KS , Geim AK, Morozov SV et al. Electric field effect in atomically thin carbon films. Science 2004; 306: 666–9. 15499015 10.1126/science.1102896

[2] Geim AK . Graphene: status and prospects. Science 2009; 324: 1530–4. 19541989 10.1126/science.1158877

[3] Mermin ND . Crystalline order in two dimensions. Phys Rev 1968; 176: 250–4.

[4] Landau LD , Lifshitz EM. Statistical Physics. Part I. Oxford: Pergamon Press, 1980.

[5] Saito R , Fujita M, Dresselhaus G et al. Physical Properties of Carbon Nanotubes. London: Imperial College London, 1998.

[6] Hoffmann R , Kabanov AA, Golov DM et al. Homo citans and carbon allotropes: for an ethics of citation. Angew Chem Int Ed 2016; 55: 10962–76. 10.1002/anie.201600655 PMC5113780 27438532

[7] Lee C , Wei X, Kysar JW et al. Measurement of the elastic properties and intrinsic strength of monolayer graphene. Science 2008; 321: 385–8. 18635798 10.1126/science.1157996

[8] Lee GH , Cooper RC, An SJ et al. High-strength chemical-vapor-deposited graphene and grain boundaries. Science 2013; 340: 1073–6. 23723231 10.1126/science.1235126

[9] Rasool HI , Ophus C, Klug WS et al. Measurement of the intrinsic strength of crystalline and polycrystalline graphene. Nat Commun 2013; 4: 2811.

[10] Yakobson BI , Brabec CJ, Bernholc J. Nanomechanics of carbon tubes: instabilities beyond linear response. Phys Rev Lett 1996; 76: 2511–4. 10060718 10.1103/PhysRevLett.76.2511

[11] Fasolino A , Los JH, Katsnelson MI. Intrinsic ripples in graphene. Nat Mater 2007; 6: 858–61. 17891144 10.1038/nmat2011

[12] Liu Y , Yakobson BI. Cones, pringles, and grain boundary landscapes in graphene topology. Nano Lett 2010; 10: 2178–83. 20481585 10.1021/nl100988r

[13] Koenig SP , Boddeti NG, Dunn ML et al. Ultrastrong adhesion of graphene membranes. Nat Nanotechnol 2011; 6: 543–6. 21841794 10.1038/nnano.2011.123

[14] Gao Y , Liu LQ, Zu SZ et al. The effect of interlayer adhesion on the mechanical behaviors of macroscopic graphene oxide papers. ACS Nano 2011; 5: 2134–41. 21341706 10.1021/nn103331x

[15] Sen D , Novoselov KS, Reis PM et al. Tearing graphene sheets from adhesive substrates produces tapered nanoribbons. Small 2010; 6: 1108–16. 20449852 10.1002/smll.201000097

[16] Shi X , Yin Q, Wei Y. A theoretical analysis of the surface dependent binding, peeling and folding of graphene on single crystal copper. Carbon 2012; 50: 3055–63.

[17] Na SR , Suk JW, Tao L et al. Selective mechanical transfer of graphene from seed copper foil using rate effects. ACS Nano 2015; 9: 1325–35. 25646863 10.1021/nn505178g

[18] Filleter T , McChesney JL, Bostwick A et al. Friction and dissipation in epitaxial graphene films. Phys Rev Lett 2009; 102: 086102. 19257757 10.1103/PhysRevLett.102.086102

[19] Lee C , Li Q, Kalb W et al. Frictional characteristics of atomically thin sheets. Science 2010; 328: 76–80. 20360104 10.1126/science.1184167

[20] Choi JS , Kim JS, Byun IS et al. Friction anisotropy-driven domain imaging on exfoliated monolayer graphene. Science 2011; 333: 607–10. 21719640 10.1126/science.1207110

[21] Guo Y , Guo W, Chen C. Modifying atomic-scale friction between two graphene sheets: a molecular-force-field study. Phys Rev B 2007; 76: 155429.

[22] Zhang Z , Kutana A, Yakobson BI. Edge reconstruction-mediated graphene fracture. Nanoscale 2015; 7: 2716–22. 25583600 10.1039/c4nr06332e

[23] An J , Voelkl E, Suk JW et al. Domain (grain) boundaries and evidence of ‘twinlike’ structures in chemically vapor deposited grown graphene. ACS Nano 2011; 5: 2433–9. 21361332 10.1021/nn103102a

[24] Huang PY , Ruiz-Vargas CS, van der Zande AM et al. Grains and grain boundaries in single-layer graphene atomic patchwork quilts. Nature 2011; 469: 389–92. 21209615 10.1038/nature09718

[25] Grantab R , Shenoy VB, Ruoff RS. Anomalous strength characteristics of tilt grain boundaries in graphene. Science 2010; 330: 946–8. 21071664 10.1126/science.1196893

[26] Wei Y , Wu J, Yin H et al. The nature of strength enhancement and weakening by pentagon–heptagon defects in graphene. Nat Mater 2012; 11: 759–63. 22751178 10.1038/nmat3370

[27] Wu J , Wei Y. Grain misorientation and grain-boundary rotation dependent mechanical properties in polycrystalline graphene. J Mech Phys Solids 2013; 61: 1421–32.

[28] Teng L , Li X, Gao H. Fracture of graphene: a review. Int J Fract 2015; 196: 1–31.

[29] Cao C , Sun Y, Filleter T. Characterizing mechanical behavior of atomically thin films: a review. J Mater Res 2014; 29: 338–47.

[30] Ivanovskii AL . Graphene-based and graphene-like materials. Russ Chem Rev 2012; 81: 571–605.

[31] Penkov O , Kim HJ, Kim HJ et al. Tribology of graphene: a review. Int J Precis Eng Manuf 2014; 15: 577–85.

[32] Akinwande D , Brennan CJ, Bunch JS et al. A review on mechanics and mechanical properties of 2D materials—graphene and beyond. Extreme Mech Lett 2017; 13: 42–77.

[33] Penev ES , Artyukhov VI, Ding F et al. Unfolding the fullerene: nanotubes, graphene and poly-elemental varieties by simulations. Adv Mater 2012; 24: 4956–76. 22893442 10.1002/adma.201202322

[34] Neto AC , Guinea F, Peres NM et al. The electronic properties of graphene. Rev Mod Phys 2009; 81: 109–62. 10.1103/PhysRevLett.97.266801 17280447

[35] Ando T . The electronic properties of graphene and carbon nanotubes. NPG Asia Mater 2009; 1: 17–21.

[36] Katsnelson MI . Graphene: carbon in two dimensions. Mater Today 2007; 10: 20–7.

[37] Neto AHC . The electronic properties of graphene. Vacuum 2007; 244: 4106–11.

[38] Allen MJ , Tung VC, Kaner RB. Honeycomb carbon: a review of graphene. Chem Rev 2010; 110: 132–45. 19610631 10.1021/cr900070d

[39] Liu Z . Graphene: from basic science to useful technology. Natl Sci Rev 2015; 2: 16.

[40] Wu ZS , Feng X, Cheng HM. Recent advances in graphene-based planar micro-supercapacitors for on-chip energy storage. Natl Sci Rev 2014; 1: 277–92.

[41] Gu X , Wei Y, Yin X et al. Phononic thermal properties of two-dimensional materials. arXiv: 1705.06156.

[42] Mermin ND , Wagner H. Absence of ferromagnetism or antiferromagnetism in one- or two-dimensional isotropic Heisenberg models. Phys Rev Lett 1966; 17: 1133–6.

[43] Peierls RE . Bemerkung über Umwandlungstemperaturen. Helv Phys Acta 1934; 7: 81–3.

[44] Landau LD . Zur Theorie der phasenumwandlungen II. Phys Z Sowjetunion 1937; 11: 26–35.

[45] Alder BJ , Wainwright TE. Phase transition in elastic disks. Phys Rev 1962; 127: 359–61.

[46] Shenderova OB , Zhirnov VV, Brenner DW. Carbon nanostructures. Crit Rev Solid State Mater Sci 2002; 27: 227–356.

[47] Kumar S , Parks DM. A comprehensive lattice-stability limit surface for graphene. J Mech Phys Solids 2016; 86: 19–41.

[48] Geim AK , Novoselov AK. The rise of graphene. Nat Mater 2007; 6: 183–91. 17330084 10.1038/nmat1849

[49] Singh V , Joung D, Zhai L et al. Graphene based materials: past, present and future. Prog Mater Sci 2011; 56: 1178–271.

[50] Pereira VM , Castro Neto AH, Peres NMR. Tight-binding approach to uniaxial strain in graphene. Phys Rev B 2009; 80: 045401.

[51] Gibson LJ , Ashby MF. Cellular Solids, Structure and Properties, 2nd edn. London: Cambridge University Press, 1997.

[52] Liu F , Ming P, Li J. Ab initio calculation of ideal strength and phonon instability of graphene under tension. Phys Rev B 2007; 76: 064120.

[53] Van Lier G , Van Alsenoy C, Van Doren V et al. Ab initio study of the elastic properties of single-walled carbon nanotubes and graphene. Chem Phys Lett 2000; 326: 181–5.

[54] Zakharchenko KV , Katsnelson MI, Fasolino A. Finite temperature lattice properties of graphene beyond the quasiharmonic approximation. Phys Rev Lett 2009; 102: 046808. 19257461 10.1103/PhysRevLett.102.046808

[55] Bhatia NM , Nachbar W. Finite indentation of an elastic membrane by a spherical indenter. Int J Non Linear Mech 1968; 3: 307–24.

[56] Brugger K . Determination of third-order elastic coefficients in crystals. J Appl Phys 1965; 36: 768–73.

[57] Cadelano E , Palla PL, Giordano S et al. Nonlinear elasticity of monolayer graphene. Phys Rev Lett 2009; 102: 235502. 19658947 10.1103/PhysRevLett.102.235502

[58] Yin H , Qi HJ, Fan F et al. Griffith criterion for brittle fracture in graphene. Nano Lett 2015; 15: 1918–24. 25692229 10.1021/nl5047686

[59] Wei Y , Wang B, Wu J et al. Bending rigidity and Gaussian bending stiffness of single-layered graphene. Nano Lett 2013; 13: 26–30. 23214980 10.1021/nl303168w

[60] Sanchez-Portal D , Artacho E, Soler JM et al. Ab initio structural, elastic, and vibrational properties of carbon nanotubes. Phys Rev B 1999; 59: 12678–88.

[61] Arroyo M , Belytschko T. Finite crystal elasticity of carbon nanotubes based on the exponential Cauchy-Born rule. Phys Rev B 2004; 69: 115415.

[62] Koskinen P , Kit OO. Approximate modeling of spherical membranes. Phys Rev B 2010; 82: 235420.

[63] Nicklow R , Wakabayashi N, Smith HG. Lattice dynamics of pyrolytic graphite. Phys Rev B 1972; 5: 4951–62.

[64] Helfrich W. Elastic properties of lipid bilayers: theory and possible experiments. Zeitschrift fur Naturforschung C 1973; 28: 693–703. 10.1515/znc-1973-11-1209 4273690

[65] Lipowsky R . The conformation of membranes. Nature 1991; 349: 475–81. 1992351 10.1038/349475a0

[66] Timoshenko S , Woinowsky-Krieger S. Theory of Plates and Shells, 2nd edn. London: McGRAW-HILL Book Company, 1959.

[67] Yu L , Ru CQ. Non-classical mechanical behavior of an elastic membrane with an independent Gaussian bending rigidity. Math Mech Solids 2017; 22: 491–501.

[68] Davini C , Favata A, Paroni R. The Gaussian stiffness of graphene deduced from a continuum model based on molecular dynamics potentials. J Mech Phys Solids 2017; 104: 96–114.

[69] Ahmadpoor F , Wang P, Huang R et al. Thermal fluctuations and effective bending stiffness of elastic thin sheets and graphene: a nonlinear analysis. J Mech Phys Solids 2017; 107: 294–319.

[70] Zhang T , Li X, Gao H. Defects controlled wrinkling and topological design in graphene. J Mech Phys Solids 2014, 67: 2–13.

[71] Zhang T , Li X, Gao H. Designing graphene structures with controlled distributions of topological defects: a case study of toughness enhancement in graphene ruga. Extreme Mech Lett 2014; 1: 3–8.

[72] Boddeti NG , Liu X, Long R et al. Graphene blisters with switchable shapes controlled by pressure and adhesion. Nano Lett 2013; 13: 6216–21. 24224793 10.1021/nl4036324

[73] Girit CO , Meyer JC, Erni R et al. Graphene at the edge: stability and dynamics. Science 2009; 323: 1705–8. 19325110 10.1126/science.1166999

[74] Voznyy O , Güçlü AD, Potasz P et al. Effect of edge reconstruction and passivation on zero-energy states and magnetism in triangular graphene quantum dots with zigzag edges. Phys Rev B Cond Matter 2012; 83: 5919–26.

[75] Zhang Z , Kutana A, Yakobson BI. Edge reconstruction-mediated graphene fracture. Nanoscale 2015; 7: 2716–22. 25583600 10.1039/c4nr06332e

[76] Berger C , Song Z, Li X et al. Electronic confinement and coherence in patterned epitaxial graphene. Science 2006; 312: 1191–6. 16614173 10.1126/science.1125925

[77] Ozyilmaz B , Jarillo-Herrero P, Efetov D et al. Electronic transport and quantum hall effect in bipolar graphene p−n−p junctions. Phys Rev Lett 2007; 99: 166804. 17995279 10.1103/PhysRevLett.99.166804

[78] Li X , Wang X, Zhang L et al. Chemically derived, ultrasmooth graphene nanoribbon semiconductors. Science 2008; 319: 1229–32. 18218865 10.1126/science.1150878

[79] Campos-delgado J , Romoherrera JM, Jia X et al. Bulk production of a new form of sp^2^ carbon: crystalline graphene nanoribbons. Nano Lett 2008; 8: 2773–8. 18700805 10.1021/nl801316d

[80] Jia X , Dresselhaus MS. Controlled formation of sharp zigzag and armchair edges in graphitic nanoribbons. Science 2009; 323: 1701–5. 19325109 10.1126/science.1166862

[81] Acik M , Chabal YJ. Nature of graphene edges: a review. Jpn J Appl Phys 2011; 50: 070101.

[82] Nakada K , Fujita M, Dresselhaus G et al. Edge state in graphene ribbons: nanometer size effect and edge shape dependence. Phys Rev B 1996; 54: 17954–61. 10.1103/physrevb.54.17954 9985930

[83] Son YW , Cohen ML, Louie SG. Energy gaps in graphene nanoribbons. Phys Rev Lett 2006; 97: 216803. 17155765 10.1103/PhysRevLett.97.216803

[84] Cançado LG , Pimenta MA, Neves BRA et al. Anisotropy of the Raman spectra of nanographite ribbons. Phys Rev Lett 2004; 93: 047403. 15323793 10.1103/PhysRevLett.93.047403

[85] Jorio A , Dresselhaus G, Dresselhaus MS. Carbon Nanotubes: Advanced Topics in the Synthesis, Structure, Properties and Applications. Berlin: Springer-Verlag, 2008.

[86] Panchakarla LS , Govindaraj A, Rao CNR. Nitrogen- and boron-doped double-walled carbon nanotubes. ACS Nano 2007; 1: 494–500. 19206671 10.1021/nn700230n

[87] Hu J , Ruan X, Chen YP. Thermal conductivity and thermal rectification in graphene nanoribbons: a molecular dynamics study. Nano Lett 2009; 9: 2730–5. 19499898 10.1021/nl901231s

[88] Ritter KA , Lyding JW. The influence of edge structure on the electronic properties of graphene quantum dots and nanoribbons. Nat Mater 2009; 8: 235–42. 19219032 10.1038/nmat2378

[89] Zhang X , Xin J, Ding F. The edges of graphene. Nanoscale 2013; 5: 2556–69. 23420074 10.1039/c3nr34009k

[90] Kim K , Lee Z, Malone BD et al. Multiply folded graphene. Phys Rev B 2011; 83: 245433.

[91] Shenoy VB , Reddy CD, Ramasubramaniam A et al. Edge-stress-induced warping of graphene sheets and nanoribbons. Phys Rev Lett 2008; 101: 245501. 19113631 10.1103/PhysRevLett.101.245501

[92] Reddy CD , Ramasubramaniam A, Shenoy VB et al. Edge elastic properties of defect-free single-layer graphene sheets. Appl Phys Lett 2009; 94: 101904.

[93] Zhao H , Min K, Aluru NR. Size and chirality dependent elastic properties of graphene nanoribbons under uniaxial tension. Nano Lett 2009; 9: 3012–5. 19719113 10.1021/nl901448z

[94] Wang H , Upmanyu M. Rippling instabilities in suspended nanoribbons. Phys Rev B 2012; 86: 205411.

[95] Lu Q , Huang R. Excess energy and deformation along free edges of graphene nanoribbons. Phys Rev B 2010; 81: 155410.

[96] Gan CK , Srolovitz DJ. First-principles study of graphene edge properties and flake shapes. Phys Rev B 2010; 81: 125445.

[97] Cranford S , Buehler MJ. Twisted and coiled ultralong multilayer graphene ribbons. Modelling Simul Mater Sci Eng 2011; 19: 054003.

[98] Landau LD , Lifshitz EM. Theory of Elasticity. Oxford: Pergamon Press, 1986.

[99] Stuart SJ , Tutei AB, Harrison JA. A reactive potential for hydrocarbons with intermolecular interactions. J Chem Phys 2000; 112: 6472–86.

[100] Plimpton SJ . Fast parallel algorithms for short-range molecular dynamics. J Comput Phys 1995; 117: 1–19.

[101] Garcia-Sanchez D , van der Zande AM, Paulo AS et al. Imaging mechanical vibrations in suspended graphene sheets. Nano Lett 2008; 8: 1399–403. 18402478 10.1021/nl080201h

[102] Lu Q , Huang R. Excess energy and deformation along free edges of graphene nanoribbons. Phys Rev B 2010; 81: 155410.

[103] Dewapriya MAN , Phani AS, Rajapakse RKND. Influence of temperature and free edges on the mechanical properties of graphene. Modelling Simul Mater Sci Eng 2013; 21: 2848–55.

[104] Li F , Cheng HM, Bai S et al. Tensile strength of single-walled carbon nanotubes directly measured from their macroscopic ropes. Appl Phys Lett 2000; 77: 3161–3.

[105] Demczyk BG , Wang YM, Cumings J et al. Direct mechanical measurement of the tensile strength and elastic modulus of multiwalled carbon nanotubes. Mater Sci Eng A 2002; 334: 173–8.

[106] Kumar S , Parks DM. Strain shielding from mechanically activated covalent bond formation during nanoindentation of graphene delays the onset of failure. Nano Lett 2015; 15: 1503–10. 25554829 10.1021/nl503641c

[107] Wang B , Wu J, Gu X et al. Stable planar single-layer hexagonal silicene under tensile strain and its anomalous Poisson's ratio. Appl Phys Lett 2014; 104: 081902.

[108] Kim K , Artyukhov VI, Regan W et al. Ripping graphene: preferred directions. Nano Lett 2012; 12: 293–7. 22149252 10.1021/nl203547z

[109] Griffith AA . The phenomena of rupture and flow in solids. Phil Trans Roy Soc 1921; 221: 163–98.

[110] Bazant ZP , Planas J. Fracture and Size Effect in Concrete and Other Quasi Brittle Materials. Boca Raton: Taylor & Francis, CRC Press, 1998.

[111] Zhang P , Ma L, Fan F et al. Fracture toughness of graphene. Nat Commun 2014; 5: 3782. 24777167 10.1038/ncomms4782

[112] Hwangbo Y , Lee CK, Kim SM et al. Fracture characteristics of monolayer cvd-graphene. Sci Rep 2015; 4: 4439. 10.1038/srep04439 PMC3963064 24657996

[113] Wei X , Xiao S, Li F et al. Comparative fracture toughness of multilayer graphenes and boronitrenes. Nano Lett 2015; 15: 689–94. 25555238 10.1021/nl5042066

[114] Jhon YI , Jhon YM, Yeom GY et al. Orientation dependence of the fracture behavior of graphene. Carbon 2014; 72: 619–28.

[115] Araujo PT , Terrones M, Dresselhaus MS. Defects and impurities in graphene-like materials. Mater Today 2012; 15: 98–109.

[116] Eletskii AV , Iskandarova IM, Knizhnik AA et al. Graphene: fabrication methods and thermophysical properties. Phys-Usp 2011; 54: 227–58.

[117] Thrower DA . The study of defects in graphite by transmission electron microscopy. In: Walker PL Jr (ed.). Chemistry and Physics of Carbon, vol. 5. New York: Marcel Dekker, 1969, 217–320.

[118] Stone AJ , Wales DJ. Theoretical studies of icosahedral C_60_ and some related species. Chem Phys Lett 1986; 128: 501–3.

[119] Terrones H , Terrones M, Hernández E et al. New metallic allotropes of planar and tubular carbon. Phys Rev Lett 2000; 84: 1716–9. 11017608 10.1103/PhysRevLett.84.1716

[120] Lusk MT , Carr LD. Nanoengineering defect structures on graphene. Phys Rev Lett 2008; 100: 175503. 18518307 10.1103/PhysRevLett.100.175503

[121] Li L , Reich S, Robertson J. Defect energies of graphite: density-functional calculations. Phys Rev B 2005; 72: 184109.

[122] Terrones M , Botello-Méndez AR, Campos-Delgado J et al. Graphene and graphite nanoribbons: morphology, properties, synthesis, defects and applications Nano Today 2010; 5: 351–72.

[123] Hashimoto A , Suenaga K, Gloter A et al. Direct evidence for atomic defects in graphene layers. Nature 2004; 430: 870–3. 15318216 10.1038/nature02817

[124] Kim K , Coh S, Kisielowski C et al. Atomically perfect torn graphene edges and their reversible reconstruction. Nat Commun 2013; 4: 2723. 24177166 10.1038/ncomms3723

[125] Warner JH , Lee GD, He K et al. Bond length and charge density variations within extended arm chair defects in graphene. ACS Nano 2013; 7: 9860–6. 24148018 10.1021/nn403517m

[126] Kotakoski J , Krasheninnikov AV, Kaiser U et al. From point defects in graphene to two-dimensional amorphous carbon. Phys Rev Lett 2011; 106: 105505. 21469806 10.1103/PhysRevLett.106.105505

[127] Lahiri J , Lin Y, Bozkurt P et al. An extended defect in graphene as a metallic wire. Nat Nanotechnol 2010; 5: 326–9. 20348912 10.1038/nnano.2010.53

[128] Jiang DE , Cooper VR, Dai S. Porous graphene as the ultimate membrane for gas separation. Nano Lett 2009; 9: 4019–24. 19995080 10.1021/nl9021946

[129] Li JCM . Disclination model of high angle grain boundaries. Surf Sci 1972; 31: 12–26.

[130] Shih KK , Li JCM. Energy of grain boundaries between cusp misorientations. Surf Sci 1975; 50: 109–24.

[131] Kleman M , Friedel J. Disclinations, dislocations, and continuous defects: a reappraisal. Rev Mod Phys 2008; 80: 61–115.

[132] Romanov AE , Kolesnikova AL. Application of disclination concept to solid structures. Prog Mater Sci 2009; 54: 740–69.

[133] Warner JH , Margine ER, Mukai M et al. Dislocation-driven deformations in graphene. Science 2012; 337: 209–12. 22798609 10.1126/science.1217529

[134] Li X , Cai W, An J et al. Large-area synthesis of high-quality and uniform graphene films on copper foils. Science 2009; 324: 1312–4. 19423775 10.1126/science.1171245

[135] Kim KS , Zhao Y, Jang H et al. Large-scale pattern growth of graphene films for stretchable transparent electrodes. Nature 2009; 457: 706–10. 19145232 10.1038/nature07719

[136] Reina A , Jia X, Ho J et al. Large area, few-layer graphene films on arbitrary substrates by chemical vapor deposition. Nano Lett 2009; 9: 30–5. 19046078 10.1021/nl801827v

[137] Park S , Ruoff RS. Chemical methods for the production of graphenes. Nat Nanotechnol 2009; 4: 217–24. 19350030 10.1038/nnano.2009.58

[138] Zhao L , Rim KT, Zhou H *et al.* The atomic-scale growth of large-area monolayer graphene on single-crystal copper substrates. arXiv: 1008.3542.

[139] Yu Q , Jauregui LA, Wu W et al. Control and characterization of individual grains and grain boundaries in graphene grown by chemical vapour deposition. Nat Mater 2011; 10: 443–9. 21552269 10.1038/nmat3010

[140] Yazyev OV , Louie SG. Electronic transport in polycrystalline graphene. Nat Mater 2010; 9: 806–9. 20729847 10.1038/nmat2830

[141] Malola S , Häkkinen H, Koskinen P. Structural, chemical, and dynamical trends in graphene grain boundaries. Phys Rev B 2010; 81: 165447.

[142] Cockayne E , Rutter GM, Guisinger NP et al. Grain boundary loops in graphene. Phys Rev B 2011; 83: 195425.

[143] Kim P . Graphene: across the border. Nat Mater 2010; 9: 792–3. 20864938 10.1038/nmat2862

[144] Biró LP , Lambin P. Grain boundaries in graphene grown by chemical vapor deposition. New J Phys 2013; 15: 035024.

[145] Seymour M , Provatas N. Structural phase field crystal approach for modeling graphene and other two-dimensional structures. Phys Rev B 2016; 93: 035447.

[146] Ophus C , Shekhawat A, Rasool HI et al. Large-scale experimental and theoretical study of graphene grain boundary structures. Phys Rev B 2015; 92: 205402.

[147] Suk JW , Mancevski V, Hao Y et al. Fracture of polycrystalline graphene membranes by in situ nanoindentation in a scanning electron microscope. Phys Status Solidi RRL 2015; 9: 564–9.

[148] Shekhawat A , Ritchie RO. Toughness and strength of nanocrystalline graphene. Nat Commun 2016; 7: 10546. 26817712 10.1038/ncomms10546 PMC4738364

[149] Sha ZD , Quek SS, Pei QX et al. Inverse pseudo Hall-Petch relation in polycrystalline graphene. Sci Rep 2015; 4: 5991. 10.1038/srep05991 PMC4125985 25103818

[150] Song Z , Artyukhov VI, Yakobson BI et al. Pseudo Hall–Petch strength reduction in polycrystalline graphene. Nano Lett 2013; 13: 1829–33. 23528068 10.1021/nl400542n

[151] Zhang T , Li X, Kadkhodaei S et al. Flaw insensitive fracture in nanocrystalline graphene. Nano Lett 2012; 12: 4605–10. 22889375 10.1021/nl301908b

[152] Lin QY , Zeng YH, Liu D et al. Step-by-step fracture of two-layer stacked graphene membranes. ACS Nano 2014; 8: 10246–51. 25256835 10.1021/nn5033888

[153] Orlikowski D , Nardelli MB, Bernholc J et al. Ad-dimers on strained carbon nanotubes: a new route for quantum dot formation? Phys Rev Lett 1999; 83: 4132–5.

[154] Lusk MT , Carr LD. Nanoengineering defect structures on graphene. Phys Rev Lett 2008; 100: 175503. 18518307 10.1103/PhysRevLett.100.175503

[155] O’Hern SC , Boutilier MS, Idrobo JC et al. Selective ionic transport through tunable subnanometer pores in single-layer graphene membranes. Nano Lett. 2014; 14: 1234–41. 24490698 10.1021/nl404118f

[156] Radha B , Esfandiar A, Wang FC et al. Molecular transport through capillaries made with atomic-scale precision. Nature 2016; 538: 222–5. 27602512 10.1038/nature19363

[157] Liu Y , Yakobson BI. Cones, pringles, and grain boundary landscapes in graphene topology. Nano Lett 2010; 10: 2178–83. 20481585 10.1021/nl100988r

[158] Bhowmick S , Waghmare UV. Anisotropy of the Stone-Wales defect and warping of graphene nanoribbons: a first-principles analysis. Phys Rev B 2010; 81: 155416.

[159] Carpio A , Bonilla LL. Periodized discrete elasticity models for defects in graphene. Phys Rev B 2008; 78: 085406.

[160] Wales DJ . Chemistry, geometry, and defects in two dimensions. ACS Nano 2014; 8: 1081–5. 24524186 10.1021/nn500645r

[161] Wang Y , Crespi VH. Theory of finite-length grain boundaries of controlled misfit angle in two-dimensional materials. Nano Lett 2017; 17: 5297–303. 28793763 10.1021/acs.nanolett.7b01641

[162] Yakobson BI Mechanical relaxation and ‘intramolecular plasticity’ in carbon nanotubes. Appl Phys Lett 1998; 72: 918–20.

[163] Ding F , Jiao K, Wu M et al. Pseudoclimb and dislocation dynamics in superplastic nanotubes. Phys Rev Lett 2007; 98: 075503. 17359035 10.1103/PhysRevLett.98.075503

[164] Lee GD , Wang CZ, Yoon E et al. Diffusion, coalescence, and reconstruction of vacancy defects in graphene layers. Phys Rev Lett 2005; 95: 205501. 16384068 10.1103/PhysRevLett.95.205501

[165] Huang JY , Chen S, Ren ZF. Kink formation and motion in carbon nanotubes at high temperatures. Phys Rev Lett 2006; 97: 075501. 17026242 10.1103/PhysRevLett.97.075501

[166] Kotakoski J , Meyer JC, Kurasch S et al. Stone-Wales-type transformations in carbon nanostructures driven by electron irradiation. Phys Rev B 2011; 83: 245420.

[167] Chen S , Ertekin E, Chrzan DC. Plasticity in carbon nanotubes: cooperative conservative dislocation motion. Phys Rev B 2010; 81: 155417.

[168] Lehtinen O , Kurasch S, Krasheninnikov AV et al. Atomic scale study of the life cycle of a dislocation in graphene from birth to annihilation. Nat Commun 2013; 4: 2098. 23812011 10.1038/ncomms3098

[169] Kurasch S , Kotakoski J, Lehtinen O et al. Atom-by-atom observation of grain boundary migration in graphene. Nano Lett 2012; 12: 3168–73. 22554303 10.1021/nl301141g

[170] Guisinger NP , Rutter GM, Crain JN et al. Atomic-scale investigation of graphene formation on 6H-SiC(0001). J Vac Sci Technol A 2008; 26: 932–7.

[171] Bunch JS , Dunn ML. Adhesion mechanics of graphene membranes. Solid State Commun 2012; 152: 1359–64.

[172] Koenig SP . Graphene membranes: mechanics, adhesion, and gas separations. Ph.D. Thesis. University of Colorado at Boulder. 2013.

[173] Peng SY , Wei YJ. On the influence of interfacial properties to the bending rigidity of layered structures. J Mech Phys Solids 2016; 92: 278–96.

[174] Lu Z , Dunn ML. Van der Waals adhesion of graphene membranes. J Appl Phys 2010; 107: 044301.

[175] Scharfenberg S , Rocklin DZ, Chialvo C et al. Probing the mechanical properties of graphene using a corrugated elastic substrate. Appl Phys Lett 2011; 98: 091908.

[176] Zong Z , Chen CL, Dokmeci MR et al. Direct measurement of graphene adhesion on silicon surface by intercalation of nanoparticles. J Appl Phys 2010; 107: 026104

[177] Yoon T , Shin WC, Kim TY et al. Direct measurement of adhesion energy of monolayer graphene as-grown on copper and its application to renewable transfer process. Nano Lett 2012; 12: 1448–52. 22335825 10.1021/nl204123h

[178] Scharfenberg S , Mansukhani N, Chialvo C et al. Observation of a snap-through instability in graphene. Appl Phys Lett 2012; 100: 021910.

[179] Cerda E , Mahadevan L. Geometry and physics of wrinkling. Phys Rev Lett 2003; 90: 074302. 12633231 10.1103/PhysRevLett.90.074302

[180] Liu X , Wang F, Wu H. Anisotropic growth of buckling-driven wrinkles in graphene monolayer. Nanotechnology 2015; 26: 065701. 25597449 10.1088/0957-4484/26/6/065701

[181] Deng B , Pang Z, Chen S et al. Wrinkle-free single-crystal graphene wafer grown on strain-engineered substrates. ACS Nano 2017; 11: 12337–45. 29191004 10.1021/acsnano.7b06196

[182] Wang Z , Devel M. Periodic ripples in suspended graphene. Phys Rev B 2011; 83: 125422.

[183] Bao W , Miao F, Chen Z et al. Controlled ripple texturing of suspended graphene and ultrathin graphite membranes. Nat Nanotechnol 2009; 4: 562–6. 19734927 10.1038/nnano.2009.191

[184] Qin Z , Taylor M, Hwang M et al. Effect of wrinkles on the surface area of graphene: toward the design of nanoelectronics. Nano Lett 2014; 14: 6520–5. 25299933 10.1021/nl503097u

[185] Tapasztó L , Dumitrică T, Kim SJ et al. Breakdown of continuum mechanics for nanometre-wavelength rippling of graphene. Nat. Phys 2012; 8: 739–42.

[186] Geim AK , Grigorieva IV. Van der Waals heterostructures. Nature 2013; 499: 419–25. 23887427 10.1038/nature12385

[187] Ruoff RS , Tersoff J, Lorents DC et al. Radial deformation of carbon nanotubes by van der waals forces. Nature 1993; 364: 514–6.

[188] Dappe YJ , Basanta MA, Flores F et al. Weak chemical interaction and van der Waals forces between graphene layers: a combined density functional and intermolecular perturbation theory approach. Phys Rev B 2006; 74: 205434.

[189] Balandin AA . Phonon engineering in graphene and van der waals materials. MRS Bull 2014; 39: 817–23.

[190] Kim Y , Cruz SS, Lee K et al. Remote epitaxy through graphene enables two-dimensional material-based layer transfer. Nature 2017; 544: 340–3. 28426001 10.1038/nature22053

[191] Dion M , Rydberg H, Schröder E et al. Van der Waals density functional for general geometries. Phys Rev Lett 2004; 92: 246401. 15245113 10.1103/PhysRevLett.92.246401

[192] Harl J , Kresse G. Accurate bulk properties from approximate many-body techniques. Phys Rev Lett 2009; 103: 056401. 19792517 10.1103/PhysRevLett.103.056401

[193] Harl J , Schimka L, Kresse G. Assessing the quality of the random phase approximation for lattice constants and atomization energies of solids. Phys Rev B 2010; 81: 115126.

[194] Grimme S , Antony J, Ehrlich S et al. A consistent and accurate ab initio parametrization of density functional dispersion correction (DFT-D) for the 94 elements H-Pu. J Chem Phys 2010; 132: 154104. 20423165 10.1063/1.3382344

[195] Grimme S , Ehrlich S, Goerigk L. Effect of the damping function in dispersion corrected density functional theory. J Comput Chem 2011; 32: 1456–65. 21370243 10.1002/jcc.21759

[196] Cooper VR . Van der Waals density functional: an appropriate exchange functional. Phys Rev B 2010; 81: 161104(R).

[197] Lee K , Murray ÉD, Kong L et al. Higher-accuracy van der Waals density functional. Phys Rev B 2010; 82: 081101.

[198] Berland K , Hyldgaard P. Exchange functional that tests the robustness of the plasmon description of the van der Waals density functional. Phys Rev B 2014; 89: 035412.

[199] Lebedeva IV , Knizhnik AA, Popov AM et al. Interlayer interaction and relative vibrations of bilayer graphene. Phys Chem Chem Phys 2011; 13: 5687–95. 21311778 10.1039/c0cp02614j

[200] Ruiz L , Xia W, Meng Z et al. A coarse-grained model for the mechanical behavior of multi-layer graphene. Carbon 2015; 82: 103–15.

[201] Zhang YY , Wang CM, Cheng Y et al. Mechanical properties of bilayer graphene sheets coupled by sp^3^ bonding. Carbon 2011; 49: 4511–7.

[202] Dappe YJ , Bolcatto PG, Ortega J et al. Dynamical screening of the van der waals interaction between graphene layers. J Phys: Condens Matter 2012; 24: 424208. 23032606 10.1088/0953-8984/24/42/424208

[203] Hamada I , Otani M. Comparative van der waals density-functional study of graphene on metal surfaces. Phys Rev B Cond Matter 2010; 82: 557.

[204] Liu Z , Yang J, Grey F et al. Observation of microscale superlubricity in graphite. Phys Rev Lett 2012; 108: 205503. 23003154 10.1103/PhysRevLett.108.205503

[205] Wang G , Dai Z, Wang Y et al. Measuring interlayer shear stress in bilayer graphene. Phys Rev Lett 2017; 119: 036101. 28777616 10.1103/PhysRevLett.119.036101

[206] David N , Tsvi P, Steven W. Statistical Mechanics of Membranes and Surfaces, 2nd edn. Singapore/River Edge, NJ: World Scientific Pub, 2004.

[207] Qin Z , Jung GS, Kang MJ et al. The mechanics and design of a lightweight three-dimensional graphene assembly. Sci Adv 2017; 3: 1601536. 10.1126/sciadv.1601536 PMC5218516 28070559

[208] Gao HL , Zhu YB, Mao LB et al. Super-elastic and fatigue resistant carbon material with lamellar multi-arch microstructure. Nat Commun 2016; 7: 12920. 27676215 10.1038/ncomms12920 PMC5052633

[209] Ma Y , Chen Y. Three-dimensional graphene networks: synthesis, properties and applications. Natl Sci Rev 2015; 2: 40–53.

[210] Huang X , Qi X, Boey F et al. Graphene-based composites. Chem Soc Rev 2011; 41: 666–86. 21796314 10.1039/c1cs15078b

[211] Zhang X , Li Q, Holesinger TG et al. Ultrastrong, stiff, and lightweight carbon-nanotube fibers. Adv Mater 2007; 19: 4198–201.

[212] Dikin DA , Stankovich S, Zimney EJ et al. Preparation and characterization of graphene oxide paper. Nature 2007; 448: 457–60. 17653188 10.1038/nature06016

[213] Kim KH , Oh Y, Islam MF. Graphene coating makes carbon nanotube aerogels superelastic and resistant to fatigue. Nat Nanotechnol 2012; 7: 562–6. 22820743 10.1038/nnano.2012.118

[214] Krainyukova NV , Zubarev EN. Carbon honeycomb high capacity storage for gaseous and liquid species. Phys Rev Lett 2016; 116: 055501. 26894716 10.1103/PhysRevLett.116.055501

[215] Pang Z , Gu X, Wei Y et al. Bottom-up design of three-dimensional carbon-honeycomb with superb specific strength and high thermal conductivity. Nano Lett 2017; 17: 179–85. 28073254 10.1021/acs.nanolett.6b03711

[216] Zhu W , Low T, Perebeinos V et al. Structure and electronic transport in graphene wrinkles. Nano Lett 2012; 12: 3431–6. 22646513 10.1021/nl300563h

[217] Vasić B , Zurutuza A, Gajić R. Spatial variation of wear and electrical properties across wrinkles in chemical vapour deposition graphene. Carbon 2016; 102: 304–10.

[218] Zhang YH , Wang B, Zhang HR et al. The distribution of wrinkles and their effects on the oxidation resistance of chemical vapor deposition graphene. Carbon 2014; 70: 81–6.

[219] Chen S , Li Q, Zhang Q et al. Thermal conductivity measurements of suspended graphene with and without wrinkles by micro-Raman mapping. Nanotechnology 2012; 23: 365701–4. 22910228 10.1088/0957-4484/23/36/365701

[220] Bunch JS , van der Zande AM, Verbridge SS et al. Electromechanical resonators from graphene sheets. Science 2007; 315: 490–3. 17255506 10.1126/science.1136836

